# On the natural spatio-temporal heterogeneity of South Pacific nitrous oxide

**DOI:** 10.1038/s41467-020-17509-6

**Published:** 2020-07-28

**Authors:** Andrew R. Babbin, Elisabeth L. Boles, Jens Mühle, Ray F. Weiss

**Affiliations:** 1 Department of Earth, Atmospheric & Planetary Sciences, Massachusetts Institute of Technology, Cambridge, MA 02139 USA; 2 Scripps Institution of Oceanography, University of California San Diego, La Jolla, CA 92093 USA; 3 Present Address: Department of Civil & Environmental Engineering, Stanford University, Stanford, CA 94305 USA

**Keywords:** Biogeochemistry, Climate sciences, Ocean sciences

## Abstract

Nitrous oxide (N_2_O) is a powerful greenhouse gas and ozone depleting substance, but its natural sources, especially marine emissions, are poorly constrained. Localized high concentrations have been observed in the oxygen minimum zones (OMZs) of the tropical Pacific but the impacts of El Niño cycles on this key source region are unknown. Here we show atmospheric monitoring station measurements in Samoa combined with atmospheric back-trajectories provide novel information on N_2_O variability across the South Pacific. Remarkable elevations in Samoan concentrations are obtained in air parcels that pass over the OMZ. The data further reveal that average concentrations of these OMZ air parcels are augmented during La Niña and decrease sharply during El Niño. The observed natural spatial heterogeneities and temporal dynamics in marine N_2_O emissions can confound attempts to develop future projections of this climatically active gas as low oxygen zones are predicted to expand and El Niño cycles change.

## Abstract (web summary)

Ocean oxygen minimum zones (OMZs) are known to emit the powerful greenhouse gas N_2_O, but global emission dynamics are not constrained. Here the authors use air trajectory analyses and find that air masses pick up N_2_O as they pass over OMZs, and that overall concentrations are elevated during La Niña events.

## Introduction

Nitrous oxide (N_2_O) is a gas of significant importance to current and future climate change. As both a potent greenhouse gas^1^ and substantial agent of stratospheric ozone destruction^2^, it serves a dual role in regulating climate. Its natural sources are largely microbial: N_2_O is a product of the nitrification and denitrification metabolisms, occurring throughout both terrestrial and marine ecosystems. Atmospheric N_2_O concentrations are currently growing, mainly because of artificial fertilizer application^3^. The secondary effects of global change, from warming oceans and circulation changes, could further increase N_2_O concentrations. Heterogeneity in sources and sinks on land and in the ocean makes constraining the global budget difficult. The ocean source exhibits a large degree of spatial structure, with N_2_O production being potentially concentrated in the confines of the oxygen minimum zones (OMZs) of the eastern tropical Pacific and Arabian Sea^4^, because N_2_O production from both microbial metabolisms is amplified under low oxygen conditions. This localization to remote areas impedes the acquisition of directly-measured data required to understand the system. Correct attribution of the dynamic sources and their mechanistic controls is key to interpreting observations and predicting future feedbacks on climate.

Global estimates of marine N_2_O emissions span a wide range due to the dearth of direct measurements at the sites of significant production within the water column. The most recent 5th IPCC Assessment Report^5^ suggests magnitudes for total natural emissions from soils, ocean, and atmosphere between 5.4 and 19.6 Tg N of N_2_O yr^−1^. Estimates for oceanic emissions alone range from 1.8 to 9.4 Tg N yr^−1^, with the upper estimate increasing with each subsequent IPCC report as more measurements are made and higher precision models developed. Much of the uncertainty regarding the marine N_2_O efflux derives from the ability of localized highly productive, and by consequence, suboxic waters to dominate the net budget.

In the global ocean, N_2_O is generated much as it is on land: certain groups of microorganisms harness inorganic forms of nitrogen for energy and release N_2_O as an inefficient byproduct. One of these sets of organisms–the nitrifiers–oxidize ammonium to nitrite and nitrate released through respiration, of which a small fraction, ~1 out of 10,000 molecules^6,7^ is typically emitted as N_2_O. This minor global marine pathway may be swamped by production localized to the OMZs. When oxygen (O_2_) concentrations are highly reduced, as occurs in the OMZs, the production pathways diversify with dramatic effects on N_2_O production. The efficiency of nitrification plummets as O_2_ is consumed, observed both in cultures^6^ and in the environment^8^, increasing the N_2_O yield to as much as 1 molecule in 50. In addition, at very low O_2_ concentrations, another microbial pathway–denitrification–is enabled, which can rapidly produce N_2_O as an obligate metabolic intermediate^9^. The high productivity stimulating N_2_O production leads to shallow depths of maximal concentration, and thereby primes these regions with the potential to disproportionately impact the global N_2_O budget well beyond the minor basal term.

**Fig. 1:**
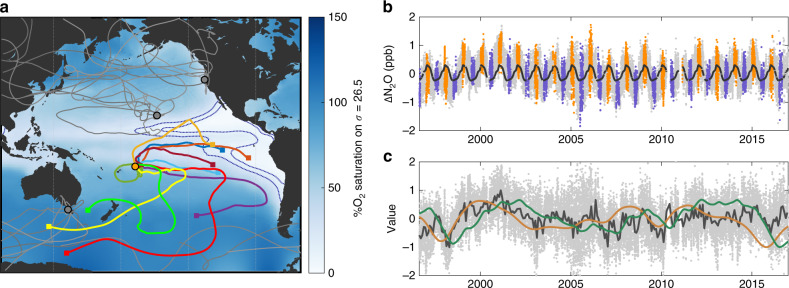
Near-continuous nitrous oxide data across the Pacific basin. **a** Spatial map of 15-day back trajectories from the four Pacific monitoring stations analyzed in this study, overlain on dissolved oxygen concentrations at the 1026.5 kg m^−3^ density horizon, with the 10 and 20% saturation contours displayed (dashed blue lines). A random selection of ten trajectories each from Hawaii, Tasmania, and California-originating back-trajectories are shown in gray, and ten randomly selected Samoa back-trajectories in color. Squares represent the positions of the trajectories 15 days prior to arrival at the Samoa station. Only the Samoa station back-trajectories frequently intersect the boundaries of the oxygen minimum zones. **b** De-trended time series of atmospheric N_2_O concentrations measured at the Samoa station, split among austral summer (orange) and winter (purple) along with a mean annual oscillation (black). This seasonal cycle is driven largely by the southward shift of the intertropical convergence zone in January bringing the influence of higher northern hemisphere concentrations to the south. **c** De-seasonalized N_2_O concentrations (light gray dots, with monthly means represented by the dark gray line) compared with a 3-month forward shifted Niño 3.4 index (orange) and a running integrated Niño 3.4 index (green). All time series are shown normalized. The Niño index time series is flipped vertically to facilitate visual comparison with N_2_O, i.e., high N_2_O aligns with negative La Niña excursions. The N_2_O concentrations align well with both ENSO metrics, with *p* < 0.0001.

OMZs are linked to strong wind-induced upwelling, which brings nutrient rich water from depth to the surface^10^, fueling high rates of primary productivity. When this newly fixed organic matter sinks into the ocean interior, it enhances biological respiration and reduces O_2_ concentrations to below 0.01% saturation^11,12^ at shallow intermediate depths of ~100–500 m. Slow lateral ventilation of these regions restricts resupply of O_2_, resulting in permanently suboxic zones and the sustenance of anaerobic microbial metabolisms^13^. These waters tend to achieve their lowest O_2_ concentrations along a fairly conserved surface-normalized density layer^14–16^, of 1026.5 kg m^−3^ (Fig. 1a). Although marine measurements of N_2_O are still fairly limited, studies have found OMZs to harbor the greatest N_2_O concentrations and fluxes by far. N_2_O supersaturations by as much as 100-fold above atmospheric equilibrium in the Eastern Tropical South Pacific (ETSP) OMZ have been observed off the coast of Peru, with estimates that this region alone could account for nearly a quarter of global marine N_2_O emissions^17^. Similar measurements and flux estimates exist for the analogous Eastern Tropical North Pacific^18^ and Arabian Sea^19^, with comparably high surface supersaturations and atmospheric emissions^20^.

The remoteness of these OMZs has hampered their direct study. Unsurprisingly then, the specialized processes of suboxic environments have yet to be fully incorporated into climate models and global budgets. Further, while OMZ regions are small on a global scale (accounting for only ~0.1% of ocean volume), the surrounding hypoxic waters are much larger (10% of ocean volume)^4^. In these hypoxic regions on the borders of OMZs where O_2_ is low, N_2_O production peaks at depths close to the surface^18^, between 50 and 200 m, which stand out as hotspots of N_2_O concentrations at depth^14,21^. The N_2_O produced by basal nitrification globally achieves maximum concentration at deeper depths^14^, between 500 and 1000 m, and is insulated from rapid atmospheric communication, even if nitrification rates peak shallower in the ocean^22^. As such, N_2_O produced in the mesopelagic deep would not be expected to display much spatial heterogeneity. In contrast, the shallower and steeper subsurface N_2_O maxima in the OMZs combine to enable rapid diffusive mixing into the surface ocean and establish these regions as hotspots of N_2_O efflux to the atmosphere. Critically, in OMZs, N_2_O exhibits low residence times and rapid turnover rates, permitting high variability, both over seasonal and interannual scales^18,23,24^ and as the OMZs expand into the future^25,26^. This feedback among OMZ size, N_2_O emissions, and climate emphasizes the importance of quantifying the N_2_O production of these regions.

Here we take a complementary approach to investigate the role of the OMZs in N_2_O emission compared with the open ocean, not by directly visiting these regions but by remotely analyzing their effect on atmospheric concentrations. Due to the short time scale of tropospheric transport relative to the ocean, these monitoring stations need not be located directly within or adjacent to the region of study. In so doing, we map N_2_O anomalies across the Pacific and, because of the time series nature of the dataset, investigate the interannual variability in association with El Niño and La Niña.

## Results and discussion

### AGAGE data for marine N_2_O studies

The network of Advanced Global Atmospheric Gases Experiment (AGAGE) stations has been quantifying greenhouse gas levels including N_2_O since the late 1970s^27^. This global network now produces high frequency, high precision measurements; the data improved substantially in the mid-1990s with the incorporation of higher precision methodologies^28^. N_2_O data from the last ~20 years are measured every 40 min (Fig. 1b, Supplementary Figs. 1 and 2) and have a precision on any individual measurement of 0.1 ppb but much higher confidence for the ensemble^28,29^. Combining these measurements with atmospheric back trajectories generated by the HYSPLIT4 model from monitoring stations (Fig. 1a, Supplementary Data 1), we map the relationship between atmospheric N_2_O concentrations and marine O_2_ content. Similar analyses have previously been conducted to investigate recent continuing sources of chlorofluorocarbons from land^30^. The marine environment is dynamic, and time-resolution is difficult to sample for many biologically-active chemical parameters requiring in situ point measurements. The AGAGE dataset, however, does not suffer the same restrictions, and the high-frequency measurements can be utilized to investigate variability in detail, including the important interannual dynamics that arise due to the influence of El Niño–Southern Oscillation (ENSO) on these regions.

**Fig. 2:**
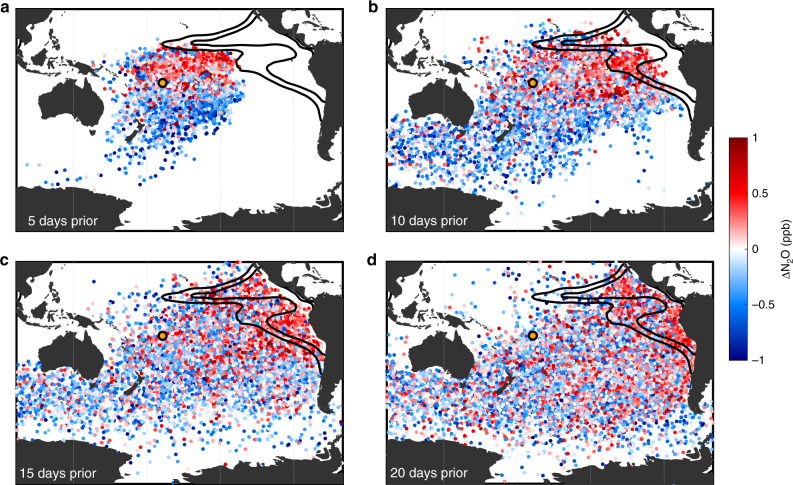
Back trajectory of Samoa atmospheric N_2_O. Locations of air parcels with their de-trended nitrous oxide measurements at Samoa are plotted (**a**) 5, (**b**) 10, (**c**) 15, and (**d**) 20 days prior to arrival at Samoa. De-trended nitrous oxide measurements are represented by the color of the corresponding dot, as anomalies relative to the station mean. The data maintain a clear spatial delineation for the full 20-day period, with much higher concentrations passing over the eastern tropical Pacific, and much lower concentrations arriving from the west and the Southern Ocean. Overlain on this map are the 10 and 20% oxygen saturation horizons at the 1026.5 kg m^−3^ potential density level (black contours). The orange circle indicates the location of the Samoa station.

**Fig. 3:**
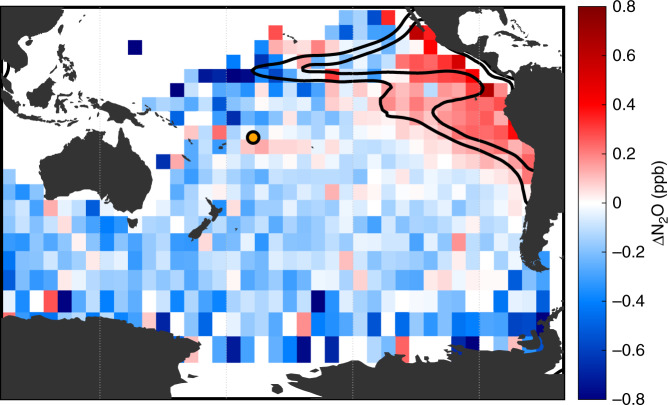
Gridded N_2_O anomalies relative to the Samoa mean. De-trended and de-seasonalized N_2_O concentrations from Samoa (color) are gridded based on back-trajectory locations 15 days prior, and a mean value is calculated for air passing over each 5° grid cell at that time (color). Overlain on this map are the 10 and 20% oxygen saturation horizons at the 1026.5 kg m^−3^ potential density level (black lines). These two data sets come from entirely independent sources, and the coincidence between them is striking; high atmospheric N_2_O concentrations align with the oxygen minimum zone, extending westward into the tropical Pacific and down the Chilean coast of South America. The orange circle indicates the location of the Samoa station.

Land sources could obscure the attribution of marine N_2_O, so all back trajectories that intersect continents over a 20-day model run are excluded in our analysis. This rigid protocol limits the choice of stations and marine areas. Multiple stations were considered for analysis, but eliminated as being ill-suited for identifying features in the OMZs. The land and monsoonal influence in the Arabian Sea obscures any marine signal in that OMZ. Similarly, the available high precision North Pacific atmospheric measurement stations are located along the California coast and in Hawaii, and do not communicate with the eastern tropical North Pacific OMZ before mixing homogenizes any advected signal (Supplementary Fig. 3). The station at American Samoa, however, permits excellent monitoring of the ETSP. The easterly trade winds rapidly transport air parcels intersecting the region of low O_2_ across the Pacific (scale of days to weeks) before turbulent mixing with other air masses is able to fully mask the OMZ signal (Fig. 2). Moreover, along this trajectory, air tends to remain in the troposphere (Supplementary Fig. 4), minimizing stratospheric impacts on surface conditions. The raw N_2_O data from Samoa, when de-trended for the long-term anthropogenic effect and de-seasonalized to minimize the intra-annual oscillation driven largely by interhemispheric transport from the north as the Intertropical Convergence Zone moves southward during boreal winter (Supplementary Figs. 1 and 2), reveal striking relationships with the ocean biogeochemical state (Fig. 3).

The seasonal modulation in Samoa N_2_O has been well-established as an effect of northern influence. However, across the Pacific sites, trajectories remain mostly well-confined to the hemispheres in which they originated (Supplementary Fig. 5). The air parcels that reach Samoa from the northern hemisphere however, do not have markedly higher N_2_O concentrations than their southern counterparts. Many high concentration trajectories do travel across the Isthmus of Panama from the Caribbean (Supplementary Fig. 6), but these are filtered out by the land filter. Notably, 20 days prior there are still a large number of high concentration trajectories in the southern hemisphere, which travel northward off the coast of South America before transiting to Samoa. Indeed, very few trajectories actually cross over the South American continent; most are deflected either north or south along its western edge. As a result, land emissions of N_2_O from South America are unlikely to have a large impact on the visible spatial signal.

### Oxygen minimum zones emerge as N_2_O hotspots

A divergence in concentrations is visible just one-day prior, with higher concentrations arriving from the north than from the south (Supplementary Fig. 3). This pattern remains moving further back in time; the highest concentrations pass over the eastern tropical Pacific, while the lowest come from the west passing over the Southern Ocean. The pattern becomes slightly less defined between 15 and 20 days-prior, as parcels with high concentrations diverge again along the western coasts of North and South America. Given the velocity of the trade winds and the distance between Samoa and the ETSP, ~8 m s^−1^ and 10^4^ km, respectively, this time scale is reasonable. Certainly the observed atmospheric concentration anomalies of N_2_O at Samoa are a combined result of intra-hemispheric biological production with interhemispheric transport, but the analysis presented herein aims to tease apart the biological impact from the tropical Pacific ocean from the additional seasonal supply from the N_2_O-enriched northern hemisphere (Supplementary Fig. 7).

In defining the OMZ boundary by the 20% O_2_ saturation contour on the 1026.5 kg m^−3^ potential density surface, the 15-day back trajectories identify higher than average N_2_O among the air parcels passing over the OMZ (Fig. 3). This isopycnal layer, generally at depths of ~200–500 m (shallower in the eastern Pacific), tends to co-locate the minimum O_2_ concentration within the water column^31^. Whereas water this deep does not tend to equilibrate rapidly with the atmosphere, this density surface reflects broader features in the water column, such as the existence of a shallower N_2_O maximum driven by microbial processes^18^. This shallower N_2_O maximum can be more easily transmitted to the surface waters from the interplay of upwelling and lateral and vertical mixing^32^. Mesoscale and sub-mesoscale eddies in particular, below the spatial resolution that can be resolved by these data, are likely pathways that promote mixing of deeper N_2_O into the surface waters^33,34^.

The air masses reaching Samoa that pass over the OMZ are ~0.4 ppb higher than the remainder of the South Pacific. This intense localization to the lowest O_2_ waters in the eastern tropical Pacific, extending southward along the Peruvian and Chilean margin, suggests a disproportionate role of OMZs and coastal upwelling waters in generating atmospheric N_2_O relative to the open ocean. Extension of higher N_2_O anomalies along the equator outside of the 20% O_2_ contour also remain visible west to 140° W (Fig. 3), albeit with reduced magnitude, indicative of a separate likely N_2_O source from equatorial upwelling and subsequent outgassing^35,36^. Further, the air overlying the coastal Pacific along the South American upwelling region is especially high in its N_2_O content, indicative of the heterogeneities that exist even within suboxic waters^17^. The coastal-most waters are the most productive and the resulting organic matter fuels greater microbial generation of N_2_O. Moreover, the depth of the anoxic onset and coincident N_2_O maximum is generally shallower toward the coast, increasing the communication between ocean and air. Narrow, shallow shelves within the OMZs in particular are important producers of N_2_O^17,23,37,38^, but local sources with such spatial resolution cannot be identified via our methods.

While the absolute difference between N_2_O mixing ratios in air that has passed over the OMZ versus those that did not is small, the abundances above the OMZs are likely much higher but are diluted to the observed anomalies after 15 days of transport and mixing. Notably, the air parcels will continue to gain N_2_O as they travel over supersaturated water masses or lose N_2_O over undersaturated areas. The coherence of the 15-day backtracked Samoan N_2_O signal and its clear demarcation with the ETSP highlight the significance of the OMZs for the marine N_2_O efflux. Indeed, the offset between the air passing over the OMZ grid cells and the rest of the South Pacific matches the average seasonal variability in the Samoan data. The seasonal cycle, which includes terrestrial sources and is heavily influenced by seasonal shifts in the intertropical convergence zone, peaks in Austral winter (January/February) with minima 0.50 ± 0.13 ppb (se) lower in summer (July/August). The rapid N_2_O cycling rates inherent to the highly productive OMZs cause these regions to respond quickly to time-dependent shifts in the underlying biogeochemistry and physical structure of the water column^18,24^.

### Further modulation by El Niño and La Niña

It is known that ETSP biogeochemistry is influenced by the El Niño/La Niña oscillations of the tropical Pacific^39,40^. La Niña brings enhanced upwelling of nutrients to the surface, thereby increasing primary production, further de-oxygenating the subsurface region, and supplying more organic matter to drive greater denitrification and N_2_O production^13^. Conversely, during an El Niño decreased upwelling reduces the surface productivity in the eastern tropical Pacific and deepens the oxycline, thereby contracting the OMZ and decreasing N_2_O emissions. In this study, the near-continuous data over 21 years permits a more quantitative analysis. The period from 1996 through 2016 included both a number of strong El Niño (1997/98, 2002/03, 2009/10, 2015/16) and La Niña (1998/99, 2007/08, 2010/11) states. Our analysis consists of dividing the N_2_O dataset among ENSO states in two different ways, both of which produced equivalent results: First, by aligning the N_2_O oscillation against the Niño 3.4 index, resulting in a time lag of 3 months relative to the ENSO state; second, by producing a running integral of the Niño 3.4 index forward in time, which essentially represents a cumulative effect of prolonged El Niño/La Niña periods (Fig. 1c). The Niño 3.4 index describes a 5-month running mean of sea surface temperature anomalies in the central equatorial Pacific, and was used because it is indicative of the broader Pacific region.

**Fig. 4:**
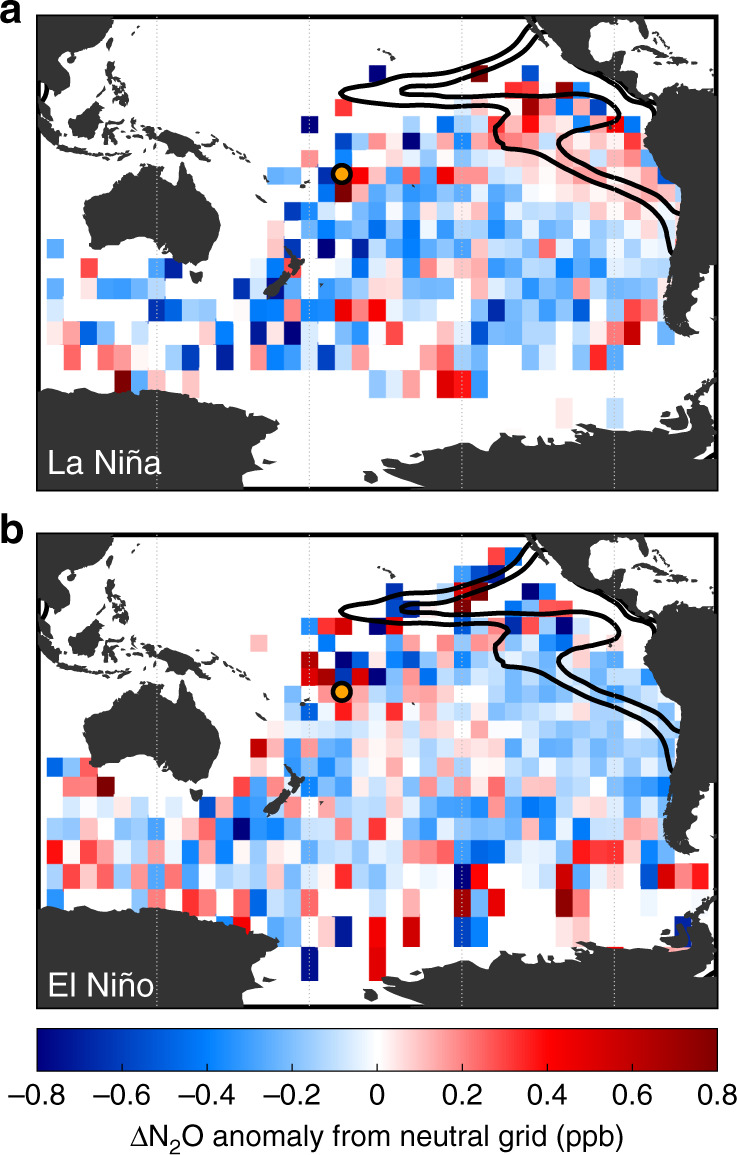
El Niño/Southern Oscillation effects on gridded N_2_O. The time series was split among three states: La Niña, with a Niño 3.4 index < −0.5; Neutral, with an index between −0.5 and 0.5; and El Niño, with an index > 0.5. New gridded maps were calculated for each, by averaging only data points within the corresponding El Niño/Neutral/La Niña states. **a** The change in each grid cell’s mean value from a Neutral state to a La Niña state. **b** The change from Neutral to El Niño. During La Niña, the eastern tropical South Pacific oxygen minimum zone (OMZ) displays heightened N_2_O in air that has passed over the water with no consistent effect in air that has passed over non-OMZ waters. During an El Niño, the concentrations in air that has passed over the OMZ are substantially reduced with again no systematic effects observed in air that has not passed over the OMZ.

**Fig. 5:**
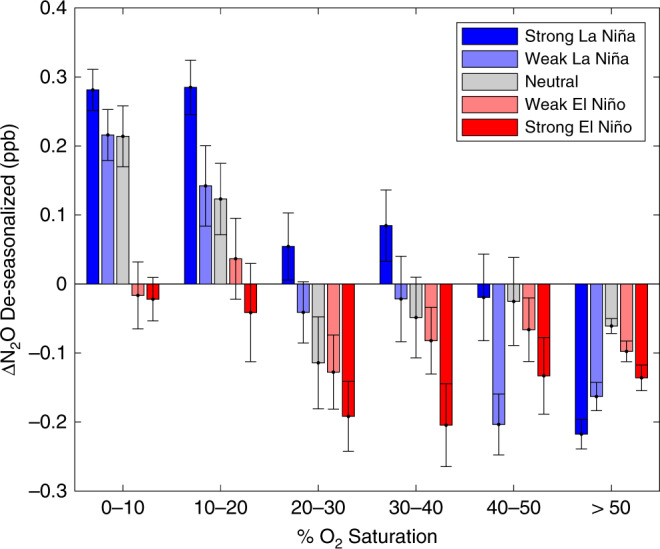
N_2_O anomalies depend on dissolved oxygen and El Niño state. Climatological oxygen concentrations are at the density level of 1026.5 kg m^−3^ and El Niño/Southern Oscillation (ENSO) state is divided by neutral (|index| < 0.5), weak (0.5 < |index| < 1.0), and strong (|index| > 1.0) states. N_2_O concentrations were separated by ENSO states and gridded as in Figs. 3 and 4, then means were calculated for grid cells of each O_2_ saturation range. N_2_O in air that has passed over the oxygen minimum zones with O_2_ < 20% saturation is greater than average during neutral and La Niña states, with highest N_2_O values at lowest % O_2_ saturation. Air parcels that have passed over regions with lowest % O_2_ saturation have nearly equal N_2_O values during strong La Niña and neutral states, but parcels that have passed over regions with 10–20% saturation exhibit substantial changes in N_2_O between the two states. Warmer surface waters across the Pacific (El Niño states), coincide with reduced N_2_O, reducing more substantially with stronger El Niño conditions, especially evident among the 0–20% O_2_ saturation waters which even show a sign change in anomaly. This El Niño effect is likely due to increased stratification of the water column isolating the deeper N_2_O rich waters from the surface. Shown are the mean ± standard error for grid cells within a bin.

Strikingly, during La Niña, air parcels passing over OMZ grid cells increase on top of their already higher than average concentrations, whereas parcels that do not pass over OMZ grid cells exhibit no systematic change (Fig. 4). This increase with La Niña is not uniform across the OMZ, however, as the lowest O_2_ waters (<10% saturation) increase by approximately one-third while those of hypoxia (<20% saturation) increase during a strong La Niña by more than double compared with neutral conditions (Table 1, Fig. 5). This occurrence implies the lowest O_2_ waters already exhibit their maximal production whereas the slightly more oxygenated waters on the periphery have potential for lower O_2_ states with La Niña to augment their N_2_O production. This decrease in O_2_ can amplify both nitrification-based production as well as expand the zone where denitrification-sourced N_2_O is permitted. During El Niño, while the parcels not passing over the OMZ do not change systematically from their mean state, those passing over the OMZ consistently see reduced N_2_O concentrations relative to their means (Table 1, Fig. 4) and lower than the long-term Samoan average. In all, the lowest O_2_ waters are by far the most variable temporally, subject to short-scale dynamics that affect these waters more than the open ocean. Such interplay confirms the importance of OMZs, with their shallow N_2_O maxima and fast generation times^18^ to marine N_2_O efflux. The cutoff of coastal upwelling associated with El Niño and the corresponding dramatic decline of primary productivity supplying organic matter to the interior curtail N_2_O production and eliminate the unique conditions required for OMZ-based production. The effects are even more pronounced during the strongest of El Niño and La Niña events, including the recent 2015-2016 El Niño, with greater shifts from the mean state during the most intense periods (Fig. 5). The Pacific-wide decrease in N_2_O observed in the AGAGE back-trajectory analysis is in line with ship-based observations from the eastern^41^, central^35^, and western^42^ Pacific basins.

**Table 1:** De-seasonalized 15-day back trajectory nitrous oxide anomalies for select spatio-temporal regimes relative to the Samoan all-time mean.

OMZ bound (% sat.)	All time points	Neutral	La Niña	El Niño
<10	0.205 ± 0.006	0.211 ± 0.010	0.290 ± 0.009	0.008 ± 0.014
<20	0.176 ± 0.005	0.174 ± 0.007	0.254 ± 0.007	0.014 ± 0.010
<30	0.144 ± 0.004	0.144 ± 0.006	0.223 ± 0.006	−0.003 ± 0.008
>30	−0.070 ± 0.003	−0.034 ± 0.005	−0.049 ± 0.007	−0.116 ± 0.005

Values are the mean ± standard error of un-gridded nitrous oxide concentrations for tracks within a bin. The oxygen minimum zone boundaries are defined by oxygen saturation at the 1026.5 kg m^−3^ density level. El Niño/Southern Oscillation states are the same as defined in Fig. 4.

ENSO impacts atmospheric circulation patterns that might obscure attribution of the observations to differences in marine production and efflux^29,43,44^. Notably, N_2_O concentrations are higher in the northern hemisphere because of greater terrestrial and anthropogenic production. Similarly, sulfur hexafluoride (SF_6_) is a compound with comparable asymmetry in hemispheric concentration because it is an industrially produced volatile compound linked with anthropogenic emissions overwhelmingly in the northern hemisphere. Indeed, SF_6_ has an even greater interhemispheric gradient and a steeper growth rate than N_2_O. In repeating our analysis for SF_6_ for which AGAGE data at Samoa are available (2005 onwards), no apparent skew toward O_2_ deficient waters (Supplementary Fig. 8) or ENSO-rooted signal arises (Supplementary Figs. 9 and 10), suggesting that the spatio-temporal relationship in N_2_O concentrations is tied to autochthonous OMZ production rather than hemispheric exchange. In completing our 15-day back trajectory analysis, very few tracks cross over the inter-tropical convergence zone, largely keeping equatorward surface winds isolated in their respective hemispheres (Supplementary Figs. 3, 6 and 7). Still, during an El Niño, there is less influence of mixing from the northern hemisphere and increased south-easterly winds, resulting in a relative decrease in atmospheric N_2_O across the southern hemisphere^45^. Indeed, this broad decrease is observed in the data (Figs. 4 and 5). Increased inter-hemispheric exchange during La Niña events should increase concentrations across the southern hemisphere, but the observed changes are to a great extent localized to air passing over the OMZs. The localization again indicates the dominance of the local biological production term over perturbations in atmospheric transport. Impacts of ENSO are not limited to the Pacific Ocean; the inter-annual variability also causes changes to terrestrial emissions of N_2_O^46,47^. Impacts of ENSO on marine biogeochemistry can be substantial sources of this interannual variation in atmospheric N_2_O^45^. Importantly, over the period of 1999–2009, El Niño periods were indeed associated with low marine N_2_O fluxes and La Niña events with high anomalies^46^.

### Summary

In developing models capable of predicting N_2_O under future climate scenarios, proper attribution of the natural sources and variability of this gas is of significant importance. The effects on these regions of anthropogenic climate change, and whether N_2_O from the OMZs indeed will increase into the future, remain open questions. Our results indicate that the variability of oceanic sources linked to OMZs can feedback substantially on the climate system as low O_2_ areas are predicted to expand^25,26^ and ENSO cycles change in intensity and frequency^48^. The enormous potential of OMZs to produce N_2_O and their direct response to El Niño/Southern Oscillation forcing, necessitates their explicit inclusion in N_2_O budget calculations and better monitoring of these regions from the eastern tropical Pacific to quantify the precise effect on N_2_O efflux before mixing reduces the local signal. The simple model presented here cannot precisely map or quantify these emissions because it does not take into account mixing or integrative effects along the transects. We also cannot directly attribute the role of transient sub-mesoscale eddies on enhanced N_2_O production^33,49,50^. While not individually observable from this dataset, such effects are encompassed in the ensemble average over the 21 years of measurements. Nonetheless, the high-frequency data from Samoa reveal not only that the ETSP is a large source of atmospheric N_2_O, but also that it is subject to such large changes in short-term budgets by ENSO that they are visible more than half-way across the Pacific Ocean.

## Methods

### Atmospheric data from AGAGE

Very few N_2_O data exist from oceanographic cruises, floats, or other ocean-going platforms, particularly from the OMZ regions of interest^51^. However, the AGAGE, maintains a network of stations around the world that have been monitoring atmospheric gases, including N_2_O, since the late 1970s. The analysis herein is limited by the locations of these stations and the frequency and precision of their measurements. Several stations around the Pacific Ocean are relevant for these inquiries into OMZs and N_2_O production: Cape Matatula in American Samoa, Trinidad Head in California, and Cape Grim in Tasmania, Australia, as well as one upper troposphere NOAA station at Mauna Loa, Hawaii. Their locations are marked on the map in Fig. 1. Some station records for N_2_O exist as far as the 1970s; however, the precision of the data was significantly improved in the mid-1990s with the implementation of new gas chromatograph multi-detector systems^28^. As a result, the analysis is limited to a recent subset of 21 years of data (1996–2016). At the AGAGE stations in the analysis, data were collected 36 times per day (a frequency of 40 min) whereas the Hawaii station logged data hourly.

To examine the overall spatial patterns and inter-annual variability in N_2_O (and sulfur hexafluoride) measurements, the long-term trend of rising anthropogenic N_2_O concentrations needs to be removed. De-trending was achieved by finding a two-degree polynomial fit and subtracting this general curve from the measured concentrations (Supplementary Fig. 1). De-seasonalization of the time series was achieved by averaging all de-trended data by month, interpolating these points to create a continuous sinusoidal seasonal cycle, and subtracting this oscillation from the de-trended series (Supplementary Fig. 2). This oscillation is largely driven by interhemispheric influence by the northern hemisphere during January/February. The de-seasonalized time series is utilized to assess interannual variability in atmospheric concentrations.

### HYSPLIT back-trajectory modeling

In order to estimate from where air parcels arriving at the monitoring stations have traveled and help determine the influence of the OMZs, NOAA’s HYSPLIT4 model was used to calculate back-trajectories from the four Pacific stations (Supplementary Fig. 3). The model uses a combination of Lagrangian and Eulerian methodologies to compute air parcel trajectories, as well as more complex dispersion, atmospheric chemistry, and deposition dynamics^52^. Climatological data was retrieved from the NCEP/DOE reanalysis project, which uses grid cells of 2.0° latitude by 1.75° longitude, and temporal coverage four times daily. In setting up the model to calculate the trajectories, the latitude, longitude, and altitude of the stations were specified. Back trajectories were calculated four times per day for 21 years, from January 1, 1996 to December 31, 2016, resulting in a total of 30,672 trajectories analyzed for each station (26,953 with associated N_2_O measurements). Each trajectory was then matched with the corresponding station’s N_2_O measurement taken nearest to the time at which the back-trajectory arrived at the station.

It is unclear how far back in time the trajectories remain valid before inaccuracies lead to severe divergences between the calculated path and the actual, and both errors from the climatological data and from the HYSPLIT model itself must be considered in evaluating the certainty of the trajectories. Generally, HYSPLIT trajectories incur a distance error on order of 20% of the track length^53^, and both errors from the climatological data and from the HYSPLIT model itself must be considered in evaluating the certainty of the trajectories. However, a large number of trajectories calculated within an ensemble, as conducted here, can minimize the influence of any individual track^54^, and the emergent patterns indicate any error to be random such that its net effect can be minimized. In order to eliminate land-based sources of emissions, a filter was created to remove all trajectories that passed over continental land within the 20-day period of calculation. For the Samoa station, the island itself was not designated as land in this filter, under the assumption that it is too small to have substantial impacts on local atmospheric N_2_O concentrations. The stations nearer to the continents (California and Tasmania) proved more difficult to filter, and local emission sources can impact the analysis results at these locations.

### Defining the ENSO strength during each trajectory

Several indices exist to measure ENSO-induced variability, and data of each is available from NOAA as monthly averages dating to 1950. Niño 3 and Niño 3.4 are now considered to be the most accurate indicators of the stage of the cycle^55^. These are based on sea surface temperature anomalies along the equator between 5° S and 5° N, from 150 to 90° W and from 170 to 120° W respectively^55^. NOAA defines an El Niño event as Niño 3.4 > 0.4, and a La Niña event as Niño 3.4 < −0.4. The Niño 1 + 2 index is measured as differences in far the eastern tropical Pacific, but has the highest variance of the indices and is not as indicative of the inter-annual cycle. The Multivariate ENSO Index version 2 (Niño MEI) was also considered; this index is the result of a combination of six variables—sea-level pressure, zonal and meridional components of the surface wind, sea surface temperature, surface air temperature, and total cloudiness fraction of the sky^56^. Niño indices also exhibit seasonal cycles, and were de-seasonalized by first subtracting monthly averages from the long-term series, then applying a Kolmogorov–Zurbenko (KZ) filter with six iterations of filters with length 2, 4, 6, 8, and 12 months to remove all variability on scales <1 year. Once de-seasonalized, the Niño 3.4 and MEI indices were very similar in phase and amplitude, so ultimately the Niño 3.4 index was used for the remainder of the analysis.

In evaluating the best metric of ENSO to use, it is also necessary to consider how biological productivity and N_2_O are expected to respond to climatic variability. It is reasonable that there should be a lag between changes in climate forcing and the biological response, though the length of lag is not apparent. Another consideration is that the biological response can be proportional to the integral of the ENSO index rather than the index itself. This supposition arises from the consideration that a long El Niño period, for example, may induce longer lasting additive impacts on OMZs and productivity than a short one, and so the cumulative state of the Pacific should be considered rather than discrete time points.

Preliminary comparisons of atmospheric data from Samoa and ENSO indices suggest that it may be necessary to incorporate this lag or integration into the analyses. The best fit for the de-seasonalized Niño 3.4 index to the Samoa data was determined to be a 3-month shift (Supplementary Fig. 5). The integral of the Niño 3.4 index, calculated with a cumulative trapezoidal numerical integral and then re-normalized, also fits the N_2_O data remarkably well (Fig. 1c). Ultimately the shifted Niño 3.4 index was chosen for analysis for simplicity and agreement with previous studies, but we note that the alignment with the integrated index implies an underlying feature of the system generating a period shift of 3 months.

### Gridding marine oxygen concentrations

Dissolved O_2_ concentrations were taken from the World Ocean Atlas 2013 with the isopycnal surfaces calculated using the TEOS-10 equation of state^16^. The O_2_ content along the isopycnal surface with *σ*_0_ = 26.5 kg m^−3^ (Figs. 2 and 4) was used to evaluate the relationship between atmospheric N_2_O and marine O_2_ content; this value is commonly taken in literature and in practice to define the depth layer of the O_2_ minimum in the Eastern Pacific^31,57^, on the order of 200–500 m below the sea surface. These subsurface O_2_ concentrations were then gridded across the Pacific in boxes of 1° × 1° latitude × longitude, and trajectory endpoints were associated with the O_2_ values of the water below them.

### Reporting summary

Further information on research design is available in the Nature Research Reporting Summary linked to this article.

## Supplementary information

Supplementary Information

Peer Review File

Supplementary Data 1

Reporting Summary

## Data Availability

All data derive from publicly accessible resources, i.e., the AGAGE network, World Ocean Atlas, and HYSPLIT model. The authors declare that the data supporting the findings of this study are available within the paper and its Supplementary Information files.
