# Supplementary material for: On the natural spatio-temporal heterogeneity of South Pacific nitrous oxide
Source: Nat Commun. 2020 Jul 28;11:3672. doi: 10.1038/s41467-020-17509-6 (PMC7387477; doi:10.1038/s41467-020-17509-6)
Supplement: Supplementary file 1 — Supplementary Information [file 41467_2020_17509_MOESM1_ESM.pdf]

Supplementary Information

On the natural spatio-temporal heterogeneity of South Pacific nitrous oxide

Andrew R. Babbin, Elisabeth L. Boles, Jens Mühle, Ray F. Weiss

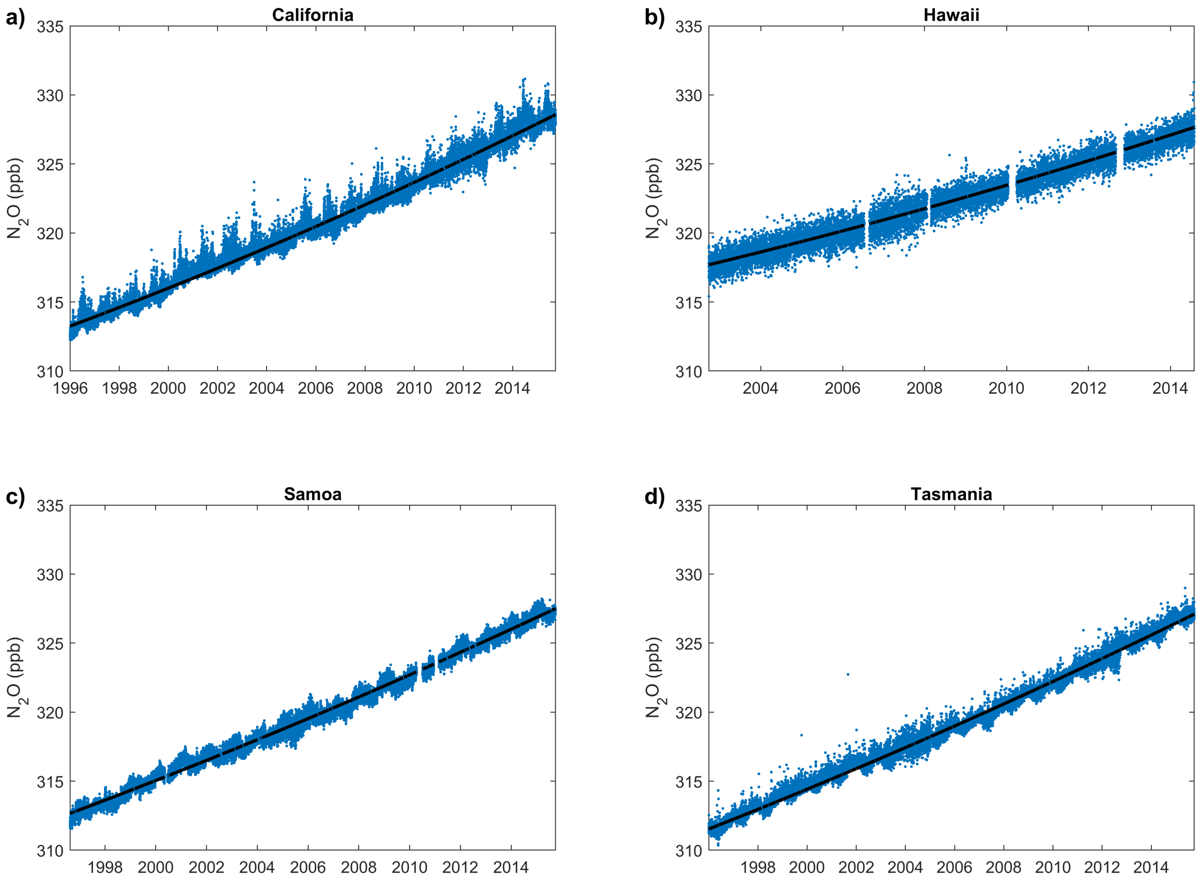

Supplementary Figure 1. Atmospheric N_2O concentrations from Pacific stations. a California, **b** Hawaii, **c** Samoa, and **d** Tasmania. The long-term trend line (black), which is subtracted from the data points to create the de-trended series, is plotted on top of data points (blue). Only the period of analysis, from 1996 to 2016, is shown. AGAGE and NOAA N_2O values are reported on slightly different calibration scales (~1 ppb differences), but this work solely uses AGAGE Samoa data for the analysis.

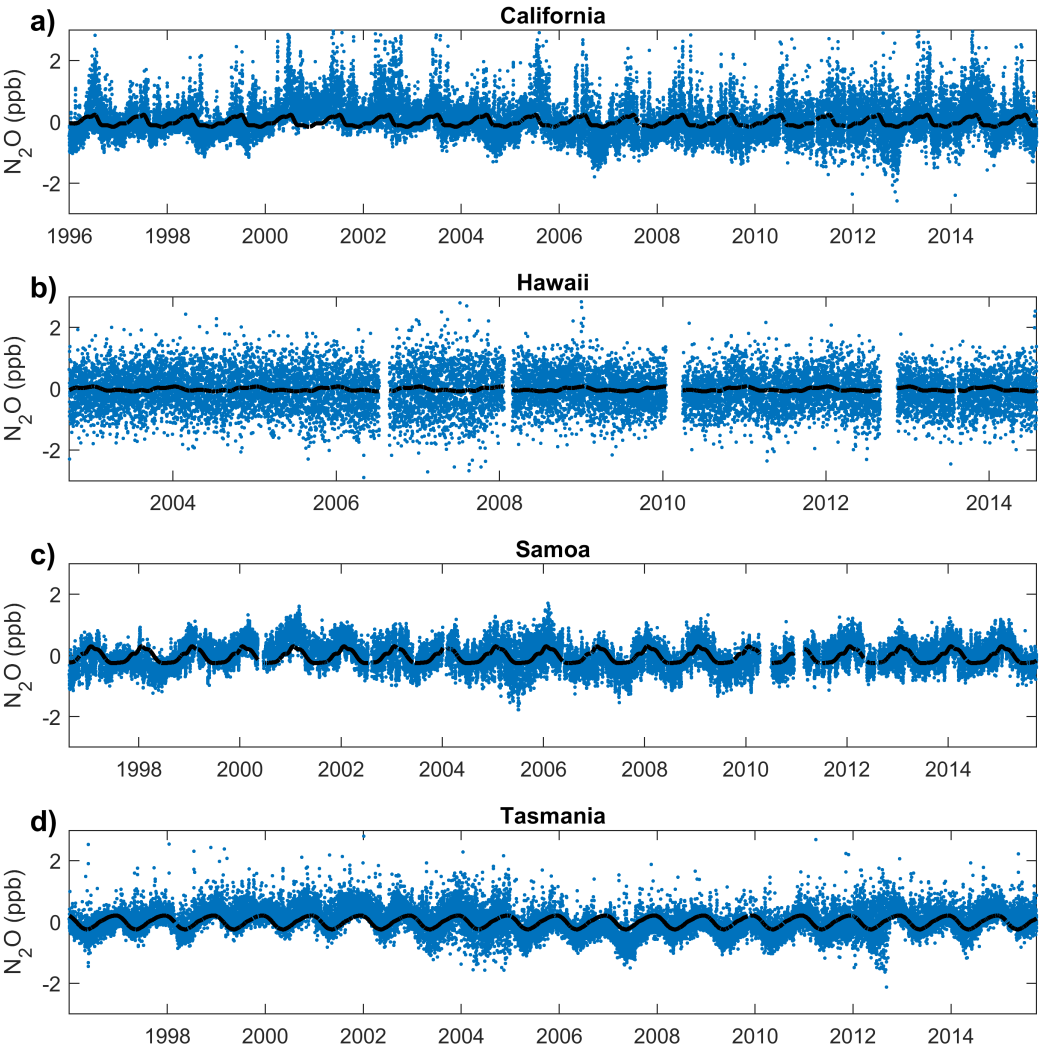

Supplementary Figure 2. De-trended atmospheric N_2O time-series from Pacific monitoring stations. a California, **b** Hawaii, **c** Samoa, and **d** Tasmania are shown. This seasonal cycle (overlaid in black) was subtracted from the de-trended data (blue) to achieve the de-seasonalized series.

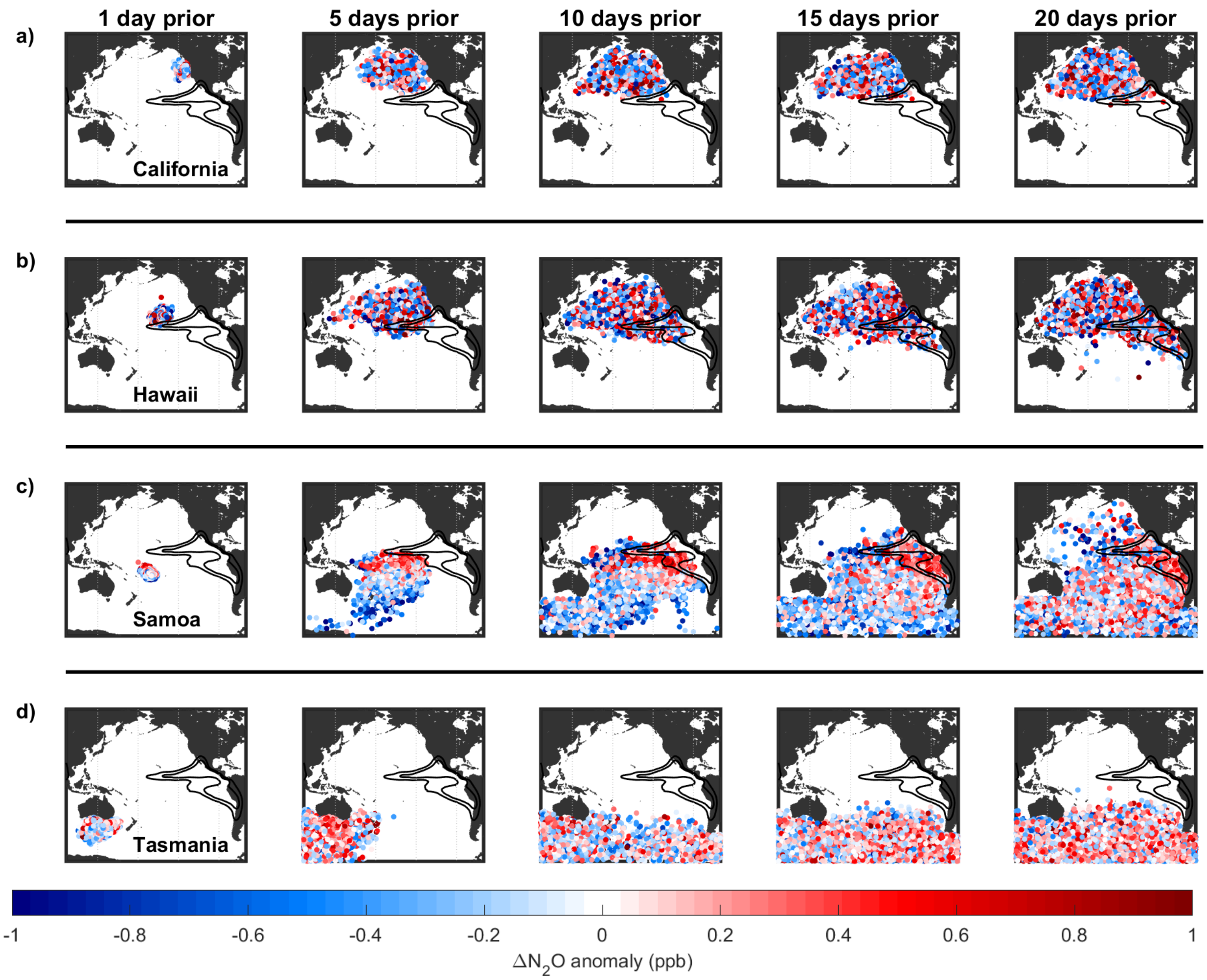

Supplementary Figure 3. Spatial trajectories from Pacific stations. **a** California, **b** Hawaii, **c** Samoa, and **d** Tasmania are shown. For each station, locations of air parcels are plotted 1, 5, 10, 15, and 20 days prior to arrival at station. All trajectories passing over land within the past 20 days have been removed. De-trended/de-seasonalized nitrous oxide measurements are represented by the color of the corresponding dot, as anomalies relative to the mean at each station. Higher than average concentrations appear red and lower than average are blue. No spatial patterns are visible in California, Hawaii, and Tasmania concentrations 5 days prior and beyond. However, Samoa data maintain a clear spatial divergence for the full 20-day period, with much higher concentrations arriving from the eastern tropical Pacific, and much lower concentrations from the west and Southern Ocean.

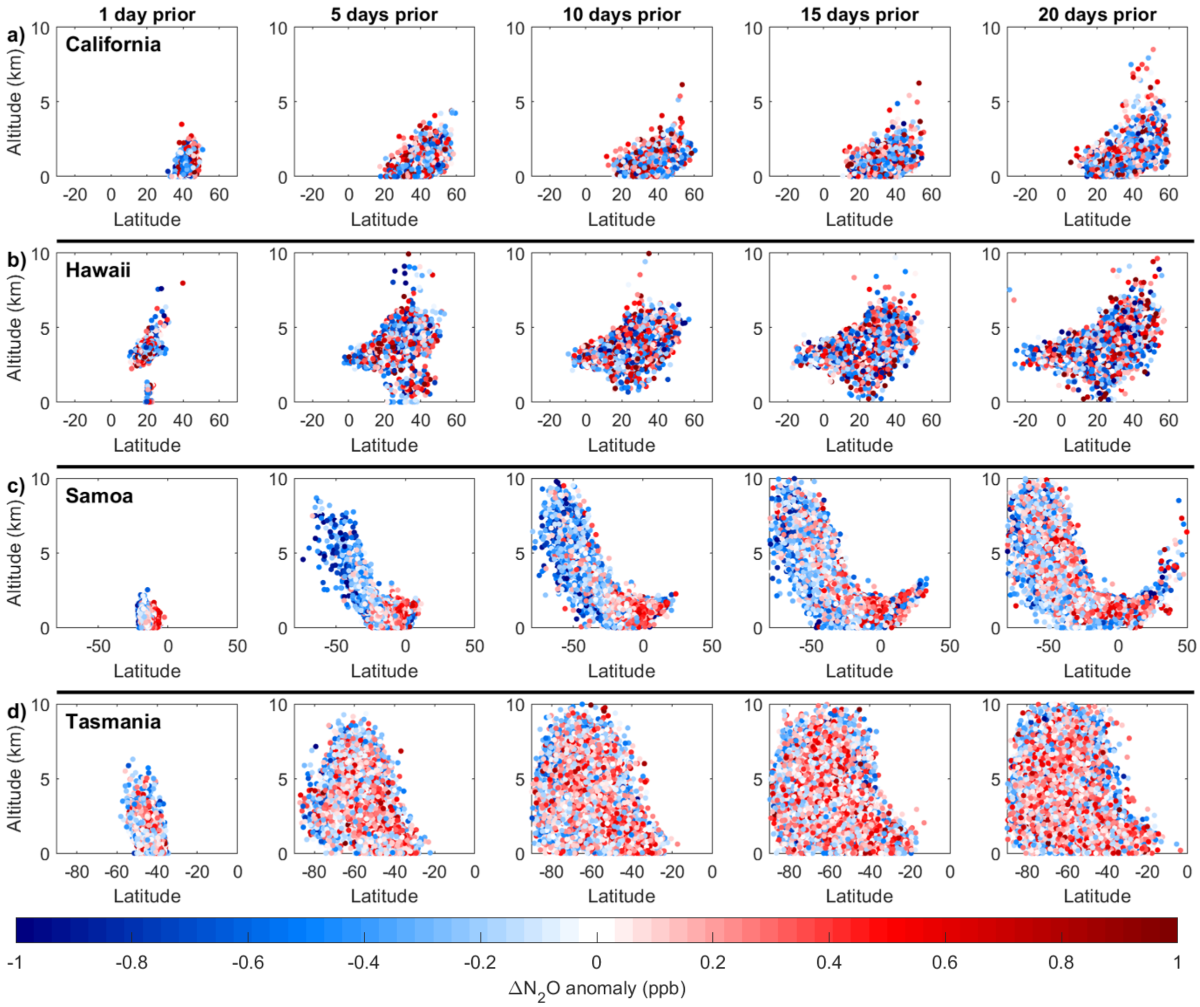

Supplementary Figure 4. Altitude trajectories and de-trended N_2O anomalies. a California, **b** Hawaii, **c** Samoa, and **d** Tasmania stations are shown. N_2O anomalies (color) are plotted versus altitude and latitude 1, 5, 10, 15 and 20-days prior to arrival at the respective station. Trajectories that pass over land are excluded. Few patterns emerge from California, Hawaii and Tasmania concentrations. Trajectories from Samoa that arrive from the north remain close to land and have high N_2O concentrations, while those arriving from farther south come from high altitudes and have low N_2O. All stations show many trajectories that reach at least 10 km altitude, which is the height of the tropopause and the top of the HYSPLIT model. At low latitudes, this pattern is a result of the Hadley Cell circulation, which brings air from high altitudes to the surface around 30° N and S latitudes, then push it equatorward. Samoa data show that the air parcels remaining nearer the surface have higher concentrations than those that come from higher altitudes, though this is not apparent for California, Hawaii, or Tasmania. Samoa is likely affected by both the northern and southern hemisphere Hadley Cells due to ITCZ shifts. The trajectories from farthest north mostly have lower concentrations than those remaining in the equatorial region 20 days prior, despite typically higher concentrations in the northern hemisphere. It is probable that the much lower concentrations from high latitude and altitude are a result of Brewer-Dobson circulation, which brings N_2O-depleted air down from the stratosphere near the poles.

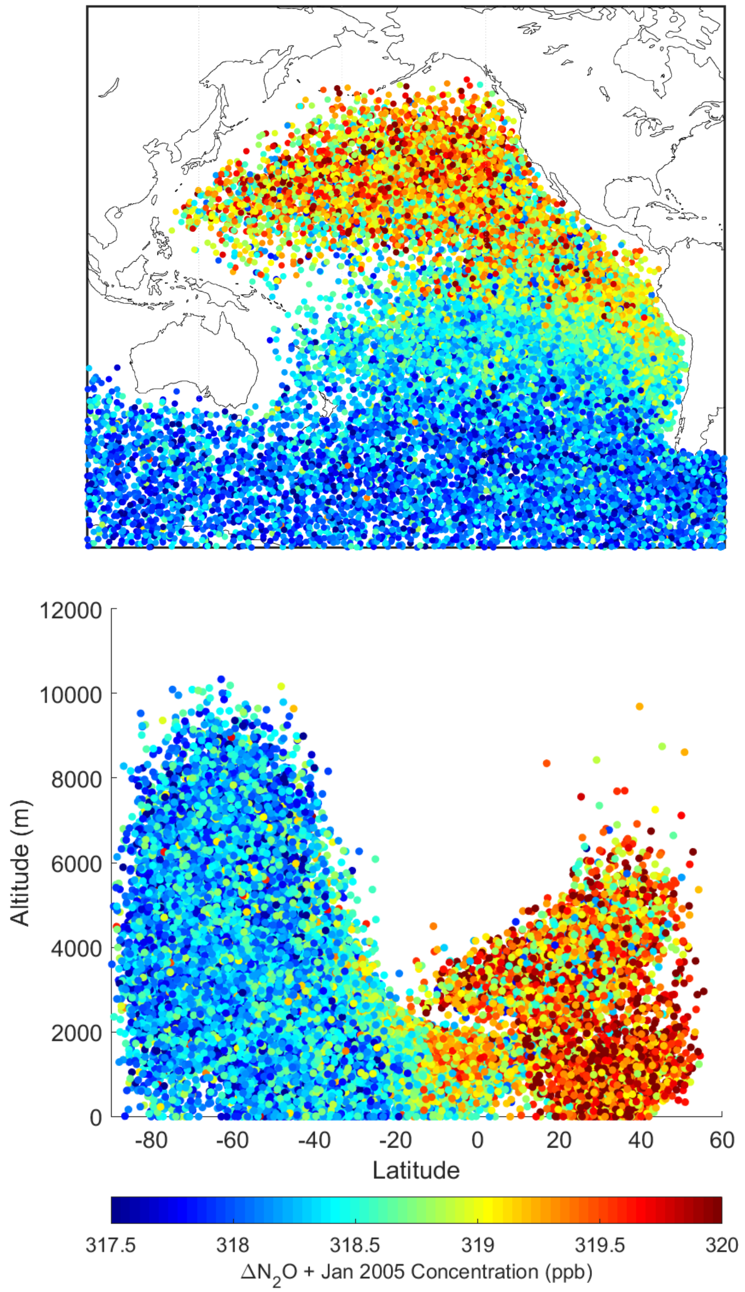

Supplementary Figure 5. 20 days prior composite trajectory positions and N_2O concentrations. Measurements from all 4 stations are shown, with all trajectories over continental land having been removed. N_2O values are de-trended, with the monthly average value from January 2005 for individual stations added back into the time series to allow for comparison among stations. **a** The northern hemisphere has much higher concentrations of N_2O than the southern hemisphere, and where interhemispheric transport occurs in the eastern Pacific, there is a gradient in concentrations. **b** However, particularly high concentrations are still visible among air parcels passing over the region near but south of the equator and below 2000 m altitude, and appear distinct from the northern hemisphere concentrations.

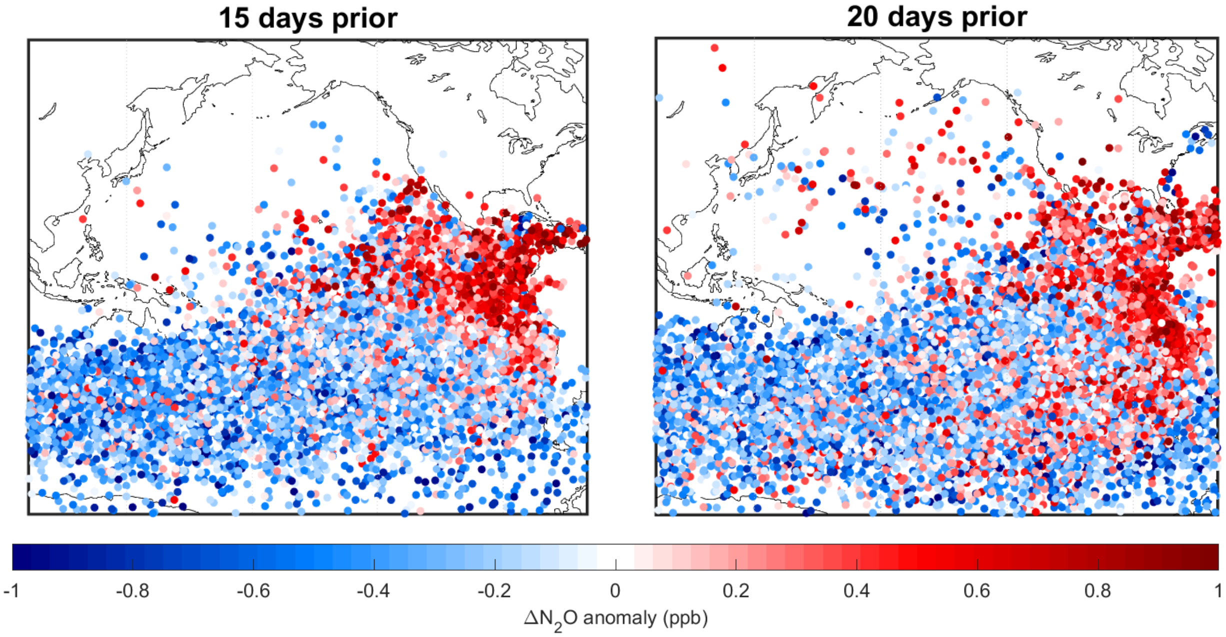

Supplementary Figure 6. Samoa back trajectories 15 and 20 days prior, including those that pass over land. Highest concentrations arrive from air that passes over the region of the Eastern Tropical Pacific. Many trajectories cross over the equator into the southern hemisphere, some traveling down the North American coast and some arriving from the Caribbean. However, interhemispheric transport does not appear to be the sole reason for high concentrations in this region, as many air parcels enriched in nitrous oxide also move northward up the west coast of South America into the tropics (visible in the 20 days prior), then ultimately move southwest to the Samoa station.

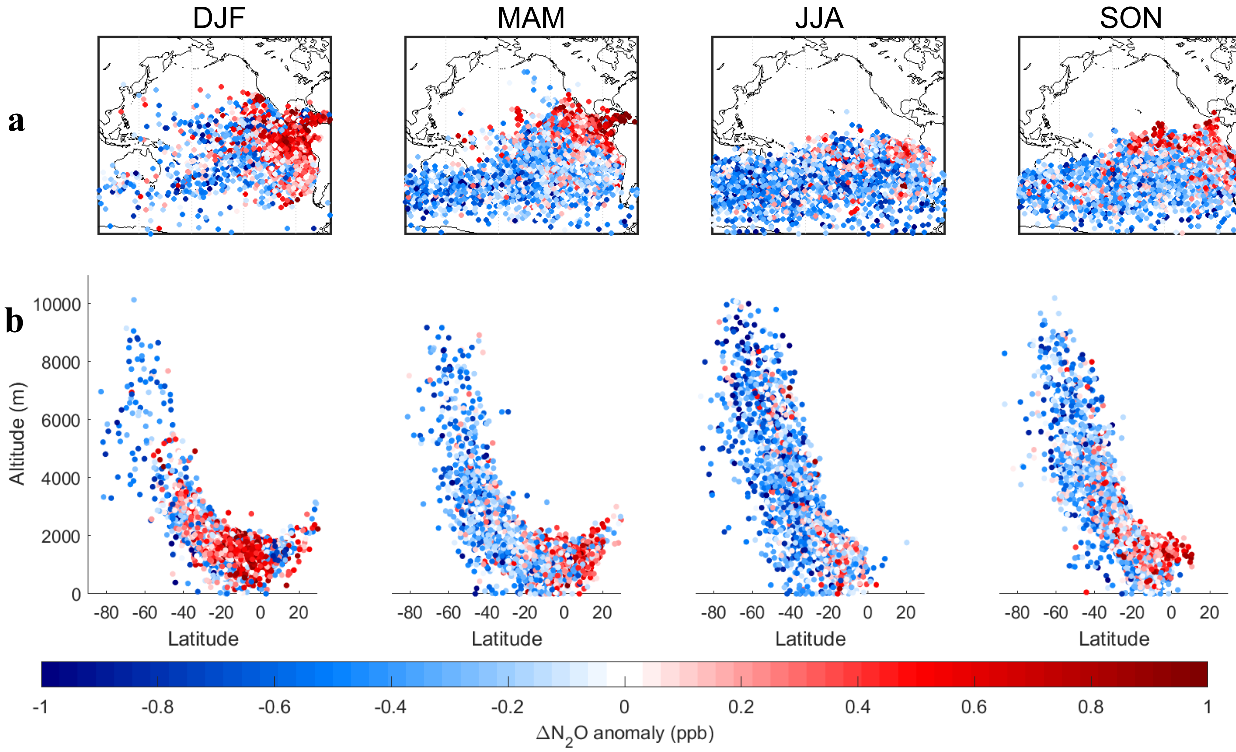

Supplementary Figure 7. Samoa back-trajectories separated by season. a Positions of all trajectories 15-days prior, revealing more transport from the eastern tropical Pacific and northern hemisphere as well as higher N_2O concentrations arriving at Samoa in austral summer and fall. **b** Positions 15-days prior plotted by altitude and longitude. In austral winter and spring, more air parcels come from the polar regions and high latitudes and arrive with lower N_2O concentrations. However, those that pass over the eastern tropical Pacific during these months still show higher than average N_2O. There is significantly more inter-hemispheric transport in the eastern Pacific during austral summer than the rest of the year; almost no inter-hemispheric transport occurs June through August. Concentrations are highest in air passing over the eastern tropical Pacific during the period when interhemispheric transport is strongest. However, the concentrations in air passing over this region still remain higher compared to air passing over the rest of the Pacific year-round, even when there is no visible interhemispheric transport, as is the case in June-August. This pattern suggests that whereas interhemispheric transport has an impact on concentrations in the southern hemisphere, more local forcing dominates the signal. It is further noteworthy that a significant number of parcels arriving from the northern hemisphere have lower than average N_2O concentrations. Especially visible from December-February with respect to altitude, the highest concentrations are in the region that spans 0–20°S, not north of the equator. The greatest N_2O emissions from the oxygen minimum zone would be anticipated December through February: in austral summer, productivity is higher in the southern hemisphere, which in turn both yields increased anoxic extent and fuels higher rates of marine N_2O production.

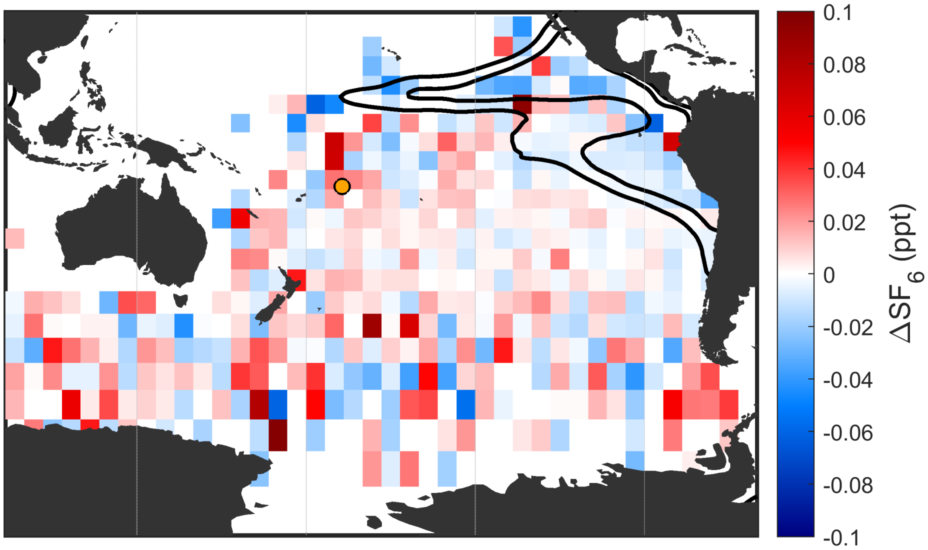

Supplementary Figure 8. Gridded mean SF_6 concentration anomalies arriving at Samoa plotted at their trajectory positions 15 days prior. The mean anomaly field (in parts per trillion) is shown relative to the Samoa (orange circle) mean. The gridding and oxygen contours are the same as shown in Fig. 3 in the main text.

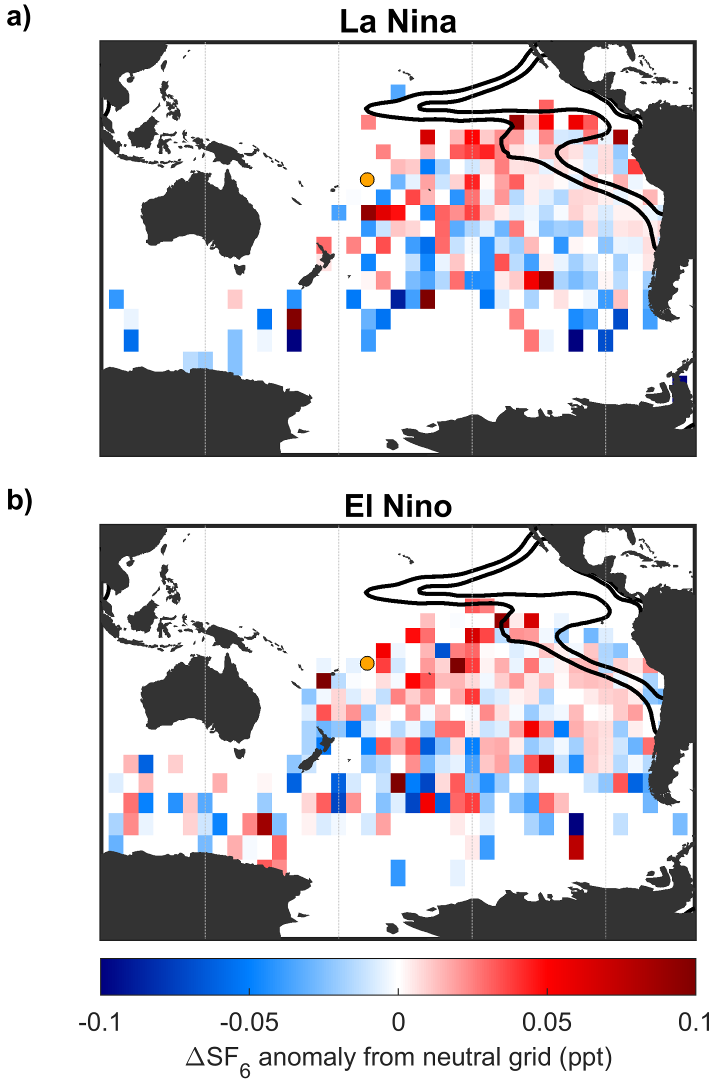

Supplementary Figure 9. Effects of ENSO on the 15-day back trajectory SF_6 concentration anomaly grid measured at Samoa. As in Fig. 4 in the main text, the time series was split between three states: La Niña, with a Niño 3.4 index < -0.5; Neutral, with an index between -0.5 and 0.5; and El Niño, with an index > 0.5. New gridded maps were calculated for each, by averaging only data points within the corresponding ENSO states. Shown in **a** is the change in each grid cell's mean value from a Neutral state to a La Niña state, and in **b** the change from Neutral to El Niño.

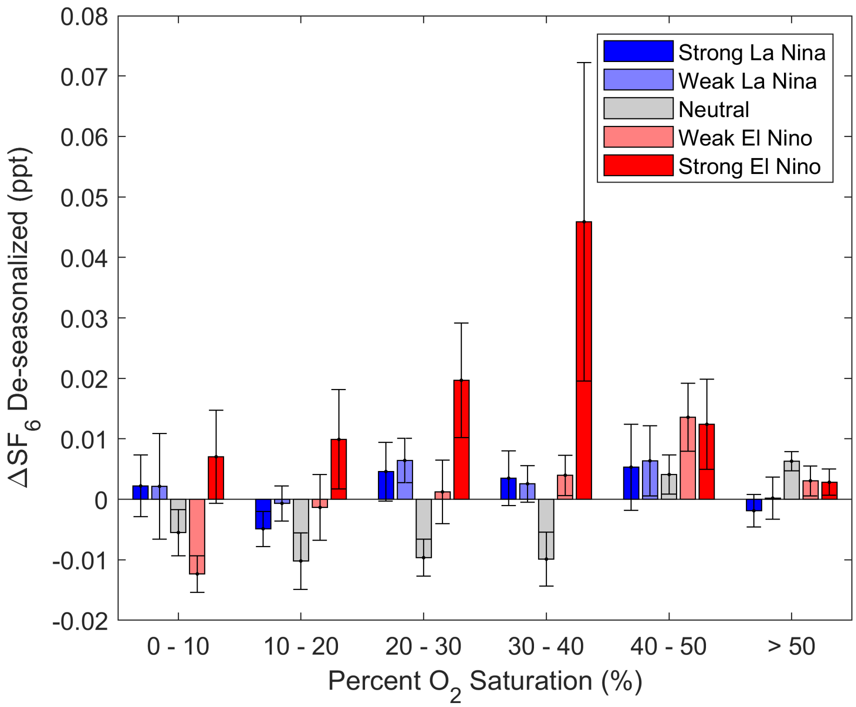

Supplementary Figure 10. Average de-seasonalized SF_6 15-day back trajectory anomalies as a function of oxygen at depth and ENSO state. Akin to Fig. 5 in the main text, oxygen concentrations are at the density level of 1026.5 kg m^{-3} and ENSO state is divided by neutral (|index| < 0.5), weak (0.5 < |index| < 1.0), and strong (|index| > 1.0) states. Unlike the histogram for N_2O, however, no systematic patterns emerge. Shown are the mean ± standard error for grid cells within a bin.
