# Supplementary material for: On the natural spatio-temporal heterogeneity of South Pacific nitrous oxide
Source: Nat Commun. 2020 Jul 28;11:3672. doi: 10.1038/s41467-020-17509-6 (PMC7387477; doi:10.1038/s41467-020-17509-6)
Supplement: Supplementary file 4 — Reporting Summary [file 41467_2020_17509_MOESM4_ESM.pdf]

nature research

Corresponding author(s): Babbin, Andrew R

Last updated by author(s): Jul 1, 2020

Reporting Summary

Nature Research wishes to improve the reproducibility of the work that we publish. This form provides structure for consistency and transparency in reporting. For further information on Nature Research policies, see our Editorial Policies and the Editorial Policy Checklist.

Statistics

For all statistical analyses, confirm that the following items are present in the figure legend, table legend, main text, or Methods section.

n/a	Confirmed	
☒	☐	The exact sample size (*n*) for each experimental group/condition, given as a discrete number and unit of measurement
☐	☒	A statement on whether measurements were taken from distinct samples or whether the same sample was measured repeatedly
☒	☐	The statistical test(s) used AND whether they are one- or two-sided<br>*Only common tests should be described solely by name; describe more complex techniques in the Methods section.*
☒	☐	A description of all covariates tested
☒	☐	A description of any assumptions or corrections, such as tests of normality and adjustment for multiple comparisons
☐	☒	A full description of the statistical parameters including central tendency (e.g. means) or other basic estimates (e.g. regression coefficient) AND variation (e.g. standard deviation) or associated estimates of uncertainty (e.g. confidence intervals)
☒	☐	For null hypothesis testing, the test statistic (e.g. *F*, *t*, *r*) with confidence intervals, effect sizes, degrees of freedom and *P* value noted<br>*Give P values as exact values whenever suitable.*
☒	☐	For Bayesian analysis, information on the choice of priors and Markov chain Monte Carlo settings
☒	☐	For hierarchical and complex designs, identification of the appropriate level for tests and full reporting of outcomes
☐	☒	Estimates of effect sizes (e.g. Cohen's *d*, Pearson's *r*), indicating how they were calculated

Our web collection on statistics for biologists contains articles on many of the points above.

Software and code

Policy information about availability of computer code

Data collection	NOAA HYSPLIT model [https://www.ready.noaa.gov/HYSPLIT.php] for atmospheric back trajectories.
Data analysis	Matlab was used for all calculations and data analysis.

For manuscripts utilizing custom algorithms or software that are central to the research but not yet described in published literature, software must be made available to editors and reviewers. We strongly encourage code deposition in a community repository (e.g. GitHub). See the Nature Research guidelines for submitting code & software for further information.

Data

Policy information about availability of data

All manuscripts must include a data availability statement. This statement should provide the following information, where applicable:

- Accession codes, unique identifiers, or web links for publicly available datasets
- A list of figures that have associated raw data
- A description of any restrictions on data availability

All data derive from publicly accessible resources, i.e., the AGAGE network, World Ocean Atlas, and HYSPLIT model. The authors declare that the data supporting the findings of this study are available within the paper and its supplementary information files.

Field-specific reporting

Please select the one below that is the best fit for your research. If you are not sure, read the appropriate sections before making your selection.

☐ Life sciences ☐ Behavioural & social sciences ☒ Ecological, evolutionary & environmental sciences

For a reference copy of the document with all sections, see nature.com/documents/nr-reporting-summary-flat.pdf

Ecological, evolutionary & environmental sciences study design

All studies must disclose on these points even when the disclosure is negative.

Study description	Nitrous oxide measurements were made at American Samoa as part of the AGAGE network, and back-tracked 15 days across the Pacific Ocean using the HYSPLIT model. Relative anomalies from the Samoan mean were plotted for all times, as well as for subsets dictated by the ENSO state.
Research sample	Primary air samples are collected 36 times per day as part of the AGAGE sampling strategy, except when instruments run standards or are down for maintenance.
Sampling strategy	All publicly available data with high precision instrumentation until the start of our analyses were used.
Data collection	Primary data come from the AGAGE monitoring station at Cape Matatula in American Samoa. Full details can be found at agage.mit.edu.
Timing and spatial scale	All data come from Cape Matatula, American Samoa over the time period January 1996 – 2016.
Data exclusions	No data were excluded.
Reproducibility	Not relevant to the work -- time series data. Although, we repeated our analysis with sulfur hexafluoride to aid in the interpretation of results related to nitrous oxide.
Randomization	Not relevant to the work -- time series data.
Blinding	Not relevant to the work -- time series data.

Did the study involve field work? ☐ Yes ☒ No

Reporting for specific materials, systems and methods

We require information from authors about some types of materials, experimental systems and methods used in many studies. Here, indicate whether each material, system or method listed is relevant to your study. If you are not sure if a list item applies to your research, read the appropriate section before selecting a response.

Materials & experimental systems

n/a	Involved in the study	
☒	☐	Antibodies
☒	☐	Eukaryotic cell lines
☒	☐	Palaeontology and archaeology
☒	☐	Animals and other organisms
☒	☐	Human research participants
☒	☐	Clinical data
☒	☐	Dual use research of concern

Methods

n/a	Involved in the study	
☒	☐	ChIP-seq
☒	☐	Flow cytometry
☒	☐	MRI-based neuroimaging
