# Supplementary material for: On the natural spatio-temporal heterogeneity of South Pacific nitrous oxide
Source: Nat Commun. 2020 Jul 28;11:3672. doi: 10.1038/s41467-020-17509-6 (PMC7387477; doi:10.1038/s41467-020-17509-6)
Supplement: Supplementary file 2 — Peer Review File [file 41467_2020_17509_MOESM2_ESM.docx]

Peer Review Information

**Journal:** Nature Communications

**Manuscript title:** On the natural spatio-temporal heterogeneity of South Pacific nitrous oxide

**Corresponding author name(s):** Dr Andrew Babbin

**Editorial notes:**

**none**

**Reviewer comments & decisions:**

**Reviewer comments, first version:**

Reviewer #1 (Remarks to the Author):

Review of Babbin et al ’On the spatio-temporal heterogeneity of South Pacific nitrous oxide’

Babbin et al present a study based on high-resolution monitoring of atmospheric nitrous oxide (N2O) concentrations, which they explore in the context of El Niño and La Niña dynamics. The study describes N2O variabilities in response to those oscillation dynamics with increased concentrations of N2O in the atmosphere during La Niña events, and decreased N2O concentrations under El Niño situations. The dataset is carefully chosen, and the methods of analysis are thoroughly described. I appreciate the new approach to explore spatio-temporal variabilities of N2O concentrations, which complements and extends cruise-based experimental approaches. For the first time, this allows to really deduct larger patterns. The dependency of N2O distribution and fluxes to the atmosphere is important to a broad community of scientists, especially and hopefully global biogeochemical modelers and political decision makers, likewise. I like this paper, I consider it a highly valuable contribution to greenhouse gas research, and I recommend publishing it in Nature Communications. The paper is well-written, the figures and tables are supportive of the explored claims, and the relevant literature is cited. I only have some minor or technical questions and remarks:


General comments:
1. I am missing a reference, which would need some discussion as it makes some different claims. I find this paper difficult to handle, however, I do think it is not an option to omit it:
Ji, Q., Altabet, M. A., Bange, H. W., Graco, M. I., Ma, X., Arévalo-Martínez, D. L., and Grundle, D. S.: Investigating the effect of El Niño on nitrous oxide distribution in the eastern tropical South Pacific, Biogeosciences, 16, 2079–2093, https://doi.org/10.5194/bg-16-2079-2019, 2019.
2. There is a mix of using abbreviations and complete names of N2O/ nitrous oxide, O2/ oxygen, OMZ/ Oxygen minimum Zone. This should be aligned throughout the manuscript.

Specific comments:
l. 21/22: the repletion of ‘air parcels that pass over the OMZ’ doesn’t sound most elegant, maybe this can be rephrased.
l. 31: N2O is not a by-product of denitrification- as you explain the processes later it could be option to just say it is a product of nitrification and denitrification
l. 34 I would like to see a reference for the fertilizer statement
l. 36 ff: Here you got to the role of OMZs for N2O budgets, for a reader not familiar with the production pathways it would be helpful to learn that low oxygen concentrations promote N2O production via both relevant pathways.
l. 195: What is the Niño 3.4 index?
l. 198: space is missing between Niño and 3.4
l. 203 ff: This is very interesting; I would like to see a discussion on the processes mainly responsible for the production from your point of view. Would this be a habitat compression leading to a combined denitrification/nitrification scenario, would it have to be mostly nitrification?
l. 204: This would be somewhat in contradiction to what Ji et al stated in the previously mentioned paper. What would be your thoughts about this, possibly superficial, difference?
l. 223: This is very elegant to prove the importance of production in the OMZ, I really appreciate this addition to the manuscript.
l. 379: The formatting for reference no 12 is weird.
In various places of the reference list numbers or signs should be in subscript/superscript.

Dear authors- this is a good paper, I enjoyed reading it!
Carolin Löscher


Reviewer #2 (Remarks to the Author):

This is the third time I have reviewed this paper, which links variability in atmospheric N2O observed at Samoa (SMO) to N2O-enriched air coming from the ETSP oxygen minimum zone (OMZ) using a back trajectory model. As before, I find the results interesting and innovative. I am persuaded that the authors are picking up a true N2O production signal associated with ventilation from the OMZ, which varies with ENSO.
My main comment at this point is that the final sentence of the abstract might be causing problems for the paper in the peer review process, “The observed natural spatial heterogeneities and temporal dynamics in marine N2O emissions can confound attempts to interpret anthropogenic sources of this climatically active gas as low oxygen zones are predicted to expand and El Niño cycles change.” This sentence is not strongly related to or supported by the body of the text, which is scientifically valuable in its own right. A similar comment could be made about the Summary statement on lines 245-256. I would rather see closing remarks that summarize the inherently interesting results presented here as opposed to a (somewhat contrived?) statement that links them to anthropogenic N2O source mitigation efforts.
A few additional comments:
Fig. 1, line 534-5. The claim that both ENSO indices align well with N2O should be supported with some correlation statistics. This is not obvious by simple visual inspection and the statement is also puzzling because the two ENSO indices are out of phase with each other.
p.12, line 278-280. In the detrending of the data shown in Figure S2, it sounds like the subtracted seasonal cycle varied year to year. Could this have affected the analysis? The seasonal cycle is influenced by shifts in wind that bring more air from the southeast during El Nino periods and more from the northeast during La Nina.
p.9, lines 203-204 “while those of hypoxia (<20% saturation) more than double on average (Table 1, Fig. 5).” Is this is comparing Neutral to La Nina conditions? If so, the N2O anomaly change is 0.136 to 0.164 ppb, which is not “more than double.” (?)
Figures 3-5 are interesting and show the data from various perspectives, but I’m also puzzled by some possible inconsistencies. For example:
Figures 4 and 5. Focusing on the OMZ 10% saturation contour area and 0-10% O2 saturation bars, respectively, Figure 4 shows the difference between La Nina and Neutral conditions to be around 0.2 ppb, while Figure 5 shows that La Nina and Neutral, “have nearly equal N2O values” (?)
Figures 3 and 5. If N2O coming from the OMZ is actually lower than the all-data mean during El Nino (negative red bars in Figure 5), why doesn’t this affect the OMZ region in Figure 3, which looks to be strongly enhanced by 0.1-0.8 ppb relative to the all-data mean?
p.8, line 192. Why wasn’t the strong El Nino of 2015-2016 included in the analysis?


Reviewer #3 (Remarks to the Author):

General Assessment
Babbin and colleagues present an atmospheric-based approach to trace the nitrous oxide (N2O) emissions from the South Pacific Ocean, with particular focus on the impact of the coastal upwelling region along the eastern boundary as well as the extensive oxygen minimum zone (OMZ). The authors aim to provide an assessment of the spatial and temporal variability on the N2O emissions from the region and how they might be affected by different phases of the ENSO. The paper is well written and the authors correctly identify relevant aspects contributing to the current uncertainties of the global budget of N2O, namely, the scarcity of data in key marine regions and the difficulty of access to remote locations. Although Babbin et al present an interesting approach, similar top-down methods have been applied before for N2O and other gases, such that from that perspective it does not represent a novel contribution. Moreover, the conclusions derived from their analysis do not significantly change our current view on the emissions from N2O from the South Pacific Ocean, i.e. beyond what has been shown already by other observational and model studies. Furthermore, considering that the study heavily relies in the description of large scale atmospheric and oceanographic features, there is little in-depth analysis of the mechanisms behind the observed trends, as well as the potential limitations of the used approach. In terms of attribution of the emissions, it is particularly critical that some of the underlying assumptions with respect to the extent and role of the OMZ were taken/interpreted from the literature without a rigorous assessment of its suitability for the purposes of supporting the atmospheric results. This, together with the omission of relevant literature with respect to ENSO in the region comes across as an oversimplification. At the end, the reader cannot clearly identify the spatial and temporal heterogeneity that is promised at the beginning of the manuscript and how it represents novel knowledge for a wider scientific community. Thus, on the grounds of lack of novelty and insufficient analysis to support the Author’s conclusions, I do not recommend this manuscript for publication in Nature Communications. In the attached document I elaborate on the aspects upon which my assessment is based.

**Author rebuttal, first version:**

Review of Babbin et al ‘On the spatio-temporal heterogeneity of South Pacific nitrous oxide’

Babbin et al present a study based on high-resolution monitoring of atmospheric nitrous oxide (N2O) concentrations, which they explore in the context of El Niño and La Niña dynamics. The study describes N2O variabilities in response to those oscillation dynamics with increased concentrations of N2O in the atmosphere during La Niña events, and decreased N2O concentrations under El Niño situations. The dataset is carefully chosen, and the methods of analysis are thoroughly described. I appreciate the new approach to explore spatio-temporal variabilities of N2O concentrations, which complements and extends cruise-based experimental approaches. For the first time, this allows to really deduct larger patterns. The dependency of N2O distribution and fluxes to the atmosphere is important to a broad community of scientists, especially and hopefully global biogeochemical modelers and political decision makers, likewise. I like this paper, I consider it a highly valuable contribution to greenhouse gas research, and I recommend publishing it in Nature Communications. The paper is well-written, the figures and tables are supportive of the explored claims, and the relevant literature is cited. I only have some minor or technical questions and remarks:

We are extremely grateful to the reviewer for these comments. We too hope it will prove a highly valuable contribution.

General comments:

1. I am missing a reference, which would need some discussion as it makes some different claims. I find this paper difficult to handle, however, I do think it is not an option to omit it:

Ji, Q., Altabet, M. A., Bange, H. W., Graco, M. I., Ma, X., Arévalo-Martínez, D. L., and Grundle, D. S.: Investigating the effect of El Niño on nitrous oxide distribution in the eastern tropical South Pacific, Biogeosciences, 16, 2079–2093, https://doi.org/10.5194/bg-16-2079-2019, 2019.

This was an oversight, and the reviewer is correct that this reference should be included; it is now referenced at **line 231**. In short, Ji et al. is an impressive study that attempts to characterize the effects of the strong 2015/16 El Niño on N_2_O production off of Peru. Their cruise in October 2015 compares that sampling to a previous occupation during a non-El Niño December 2012 sampling. The work is quite interesting, and they do see a large (75%-95%) reduction in measured N_2_O efflux from the surface. We view our study as complementary to this study: whereas they are able to resolve the shelf v. deeper OMZ for these two occupations, our longer term continuous record permits more ENSO events to be analyzed.

2. There is a mix of using abbreviations and complete names of N2O/ nitrous oxide, O2/ oxygen, OMZ/ Oxygen minimum Zone. This should be aligned throughout the manuscript.

Changed throughout, thank you! We left in three “nitrous oxide” because of the references thereafter, to avoid having N_2_O*^x^*.

Specific comments:

l. 21/22: the repletion of ‘air parcels that pass over the OMZ’ doesn’t sound most elegant, maybe this can be rephrased.

Thank you for pointing this out. We have changed the second sentence to say: “The data further reveal that average concentrations of **these OMZ air parcels** are augmented during La Niña **and decrease sharply** during El Niño.” [**line 23**].

l. 31: N2O is not a by-product of denitrification- as you explain the processes later it could be option to just say it is a product of nitrification and denitrification

This is of course correct. We have changed the text accordingly.

l. 34 I would like to see a reference for the fertilizer statement

The current best one, cited in the IPCC AR5, is: Alfi Syakila & Carolien Kroeze (2011) The global nitrous oxide budget revisited, *Greenhouse Gas Measurement and Management*, 1:1, 17-26, 10.3763/ghgmm.2010.0007. This is now included. [**line 35**].

l. 36 ff: Here you got to the role of OMZs for N2O budgets, for a reader not familiar with the production pathways it would be helpful to learn that low oxygen concentrations promote N2O production via both relevant pathways.

Good point; the sentence now reads: “The ocean source exhibits a large degree of spatial structure, with N_2_O production being potentially concentrated in the confines of the oxygen minimum zones (OMZs) of the eastern tropical Pacific and Arabian Sea^4^**, because N_2_O production from both microbial metabolisms is amplified under low oxygen conditions.**” [**line 40**].

l. 195: What is the Niño 3.4 index?

Apologies for not explaining this. The sentence now reads: “The Niño 3.4 index **describes a five-month running mean of sea surface temperature anomalies in the central equatorial Pacific, and** was used because it is indicative of the broader Pacific **region**.” [**line 208**].

l. 198: space is missing between Niño and 3.4

Edited.

l. 203 ff: This is very interesting; I would like to see a discussion on the processes mainly responsible for the production from your point of view. Would this be a habitat compression leading to a combined denitrification/nitrification scenario, would it have to be mostly nitrification?

This is a very exciting question posed by the reviewer. One could imagine all routes of production being enhanced during a La Niña on the boundaries of the OMZ, but due to the higher oxygen concentrations, between 10-20%, one could imagine nitrification is a likelier player. This said, the exact oxygen concentrations that would enable denitrification to N_2_O but not to N_2_ remain an open question. We previously showed in Babbin et al. 2015 that at ~10m layer at the oxic/anoxic transition is the sweet spot for N_2_O production by both nitrification and denitrification, and given the steepness of the oxycline, that layer can reach up to 10% oxygen saturation. As such, if the 20% saturated water (in a climatological sense) was reduced to 10% or less, both pathways could be responsible. It remains exciting fodder for future work! We now allude to this by adding the sentence, “**This decrease in O_2_ can amplify both nitrification-based production as well as expand the zone where denitrification-sourced N_2_O is permitted.**” [**line 218**].

l. 204: This would be somewhat in contradiction to what Ji et al stated in the previously mentioned paper. What would be your thoughts about this, possibly superficial, difference?

We would prefer to limit the comparison between these two studies related to effects during La Niña as the Ji et al. study had one comparison point between their El Niño and a weak La Niña, and the site was exceedingly shallow and close to shore. We’re left with the unsatisfying claim that more in situ data are required to craft a systematic view. That said, given that the very shallow coast can operate in a unique fashion with ammonium efflux from sediments, ample metals, and possible sulfide, one can certainly imagine a counter-intuitive result from such a system.

l. 223: This is very elegant to prove the importance of production in the OMZ, I really appreciate this addition to the manuscript.

Thank you, we agree!

l. 379: The formatting for reference no 12 is weird.

Indeed, thank you! For some reason, our reference manager decided to duplicate our first names.

In various places of the reference list numbers or signs should be in subscript/superscript.

We have done a thorough review to ensure appropriate baselines throughout, thank you.

Dear authors- this is a good paper, I enjoyed reading it!

Carolin Löscher

Thank you!

**Reviewer #2**

This is the third time I have reviewed this paper, which links variability in atmospheric N2O observed at Samoa (SMO) to N2O-enriched air coming from the ETSP oxygen minimum zone (OMZ) using a back trajectory model. As before, I find the results interesting and innovative. I am persuaded that the authors are picking up a true N2O production signal associated with ventilation from the OMZ, which varies with ENSO. My main comment at this point is that the final sentence of the abstract might be causing problems for the paper in the peer review process, “The observed natural spatial heterogeneities and temporal dynamics in marine N2O emissions can confound attempts to interpret anthropogenic sources of this climatically active gas as low oxygen zones are predicted to expand and El Niño cycles change.” This sentence is not strongly related to or supported by the body of the text, which is scientifically valuable in its own right. A similar comment could be made about the Summary statement on lines 245-256. I would rather see closing remarks that summarize the inherently interesting results presented here as opposed to a (somewhat contrived?) statement that links them to anthropogenic N2O source mitigation efforts.

We certainly thank the reviewer for taking yet another thorough look at this manuscript! We have edited the last sentence of the abstract to read: “The observed natural spatial heterogeneities and temporal dynamics in marine N_2_O emissions can confound attempts to **develop future projections** of this climatically active gas as low oxygen zones are predicted to expand and El Niño cycles change.” [**line 25**]

We further modified the summary statement: “In developing **models capable of predicting N_2_O under future climate scenarios, proper attribution of the natural sources and variability of this gas is of significant importance. The effects on these regions of anthropogenic climate change, and whether N_2_O from the OMZs indeed will increase into the future, remain open questions**” [**line 260**]

A few additional comments:

Fig. 1, line 534-5. The claim that both ENSO indices align well with N2O should be supported with some correlation statistics. This is not obvious by simple visual inspection and the statement is also puzzling because the two ENSO indices are out of phase with each other.

A thousand apologies! We had done these statistics previously, *p* < 0.0001, but it was an oversight not to include those in the caption here. We have now done so on **line 538**. We calculated Robust Pearson’s r correlation statistics for both metrics: they are *r* = -0.366 (shifted) and *r* = -0.521 (integrated). Despite the better fit with the integrated index, we continued forward with the shifted one for similarity to previous work in the field. For the reviewers’ benefit, the fits are shown below.

**Fig. A. Robust least squares regression between the monthly ENSO indices and the monthly average de-seasonalized N_2_O.** The integrated index is shown on the left and the shifted on the right. Both fits are highly significant, with *p* < 0.0001.

p.12, line 278-280. In the detrending of the data shown in Figure S2, it sounds like the subtracted seasonal cycle varied year to year. Could this have affected the analysis? The seasonal cycle is influenced by shifts in wind that bring more air from the southeast during El Nino periods and more from the northeast during La Nina.

It was the same seasonal oscillation that was subtracted off every year. The word “further” has been removed as it implies there were two types of de-seasonalization, which there were not. [**line 297**]

p.9, lines 203-204 “while those of hypoxia (<20% saturation) more than double on average (Table 1, Fig. 5).” Is this is comparing Neutral to La Nina conditions? If so, the N2O anomaly change is 0.136 to 0.164 ppb, which is not “more than double.” (?)

We were not very clear here! The more than double was referring to all states, but that’s not the best comparison to make. As the reviewer points out, comparing to a Neutral state is more appropriate. We have modified the sentence to read: “…**increase during a strong La Niña by more than 80% compared to neutral conditions**” [**line 215**]

Figures 3-5 are interesting and show the data from various perspectives, but I’m also puzzled by some possible inconsistencies. For example:

Figures 4 and 5. Focusing on the OMZ 10% saturation contour area and 0-10% O2 saturation bars, respectively, Figure 4 shows the difference between La Nina and Neutral conditions to be around 0.2 ppb, while Figure 5 shows that La Nina and Neutral, “have nearly equal N2O values” (?)

We see what the reviewer is suggesting with this comparison, but within this zone, La Niñas average a 0.25 ppb anomaly to the Samoa mean, compared to a 0.23 ppb anomaly during a Neutral state (Table 1). Whereas there are quite a few grid cells that appear to be much higher than that as the reviewer notes, especially within the core of the ETSP, there are a few along the equator and just north that show an opposite trend, which negate that effect. We have opted to be conservative in our interpretation of these fine-scale features, however.

Figures 3 and 5. If N2O coming from the OMZ is actually lower than the all-data mean during El Nino (negative red bars in Figure 5), why doesn’t this affect the OMZ region in Figure 3, which looks to be strongly enhanced by 0.1-0.8 ppb relative to the all-data mean?

This is a great question! It implies that the high positive values observed during a La Niña and during neutral states far outweigh the negative anomaly during an El Niño. It also appears that strong El Niños are the great equalizer in causing N_2_O to be routinely low across the entire South Pacific.

p.8, line 192. Why wasn’t the strong El Nino of 2015-2016 included in the analysis?

It was! Although it was still occurring when our dataset terminated in October 2015. The 2015 El Niño has been mentioned with the rest, **Line 203**.

**Reviewer #3**

**General Assessment**

Babbin and colleagues present an atmospheric-based approach to trace the nitrous oxide (N2O) emissions from the South Pacific Ocean, with particular focus on the impact of the coastal upwelling region along the eastern boundary as well as the extensive oxygen minimum zone (OMZ). The authors aim to provide an assessment of the spatial and temporal variability on the N2O emissions from the region and how they might be affected by different phases of the ENSO. The paper is well written and the authors correctly identify relevant aspects contributing to the current uncertainties of the global budget of N2O, namely, the scarcity of data in key marine regions and the difficulty of access to remote locations. Although Babbin et al present an interesting approach, similar top-down methods have been applied before for N2O and other gases, such that from that perspective it does not represent a novel contribution. Moreover, the conclusions derived from their analysis do not significantly change our current view on the emissions from N2O from the South Pacific Ocean, i.e. beyond what has been shown already by other observational and model studies. Furthermore, considering that the study heavily relies in the description of large scale atmospheric and oceanographic features, there is little in-depth analysis of the mechanisms behind the observed trends, as well as the potential limitations of the used approach. In terms of attribution of the emissions, it is particularly critical that some of the underlying assumptions with respect to the extent and role of the OMZ were taken/interpreted from the literature without a rigorous assessment of its suitability for the purposes of supporting the atmospheric results. This, together with the omission of relevant literature with respect to ENSO in the region comes across as an oversimplification. At the end, the reader cannot clearly identify the spatial and temporal heterogeneity that is promised at the beginning of the manuscript and how it represents novel knowledge for a wider scientific community. Thus, on the grounds of lack of novelty and insufficient analysis to support the Author’s conclusions, I do not recommend this manuscript for publication in Nature Communications. In the attached document I elaborate on the aspects upon which my assessment is based.

We appreciate the reviewer’s feedback but maintain that the use of atmospheric chemistry data measured in Samoa over 20 years to assess the pattern of N_2_O across the eastern Pacific is a unique contribution. We agree that a number of the conclusions have been suggested previously, but note that we present a dataset that can (1) ground truth the above-mentioned models severely lacking in data and (2) provide broader context for observational studies. The vast amount of data offered by atmospheric measurement greatly complements the necessary but limited *in situ* point oceanographic measurements. We respond more thoroughly to the detailed assessment below.

**General Assessment [PDF attachment]**

Babbin and colleagues present an atmospheric-based approach to trace the nitrous oxide (N2O) emissions from the South Pacific Ocean, with particular focus on the impact of the coastal upwelling region along the eastern boundary as well as the extensive oxygen minimum zone (OMZ). The authors aim to provide an assessment of the spatial and temporal variability on the N2O emissions from the region and how they might be affected by different phases of the ENSO. The paper is well written and the authors correctly identify relevant aspects contributing to the current uncertainties of the global budget of N2O, namely, the scarcity of data in key marine regions and the difficulty of access to remote locations. Although Babbin et al present an interesting approach, similar top-down methods have been applied before for N2O and other gases, such that from that perspective it does not represent a novel contribution. Moreover, the conclusions derived from their analysis do not significantly change our current view on the emissions from N2O from the South Pacific Ocean, i.e. beyond what has been shown already by other observational and model studies. Furthermore, considering that the study heavily relies in the description of large scale atmospheric and oceanographic features, there is little in-depth analysis of the mechanisms behind the observed trends, as well as the potential limitations of the used approach. In terms of attribution of the emissions, it is particularly critical that some of the underlying assumptions with respect to the extent and role of the OMZ were taken/interpreted from the literature without a rigorous assessment of its suitability for the purposes of supporting the atmospheric results. This, together with the omission of relevant literature with respect to ENSO in the region comes across as an oversimplification. At the end, the reader cannot clearly identify the spatial and temporal heterogeneity that is promised at the beginning of the manuscript and how it represents novel knowledge for a wider scientific community. Thus, on the grounds of lack of novelty and insufficient analysis to support the Author’s conclusions, I do not recommend this manuscript for publication in Nature Communications.

We are truly appreciative of the time and effort the reviewer dedicated to thoroughly reviewing our manuscript. We were not as clear as we could have been in parts, when expressing what indeed is new, either by approach, or in the knowledge generated. We hope our responses to the points below clarify our choices made. Regarding novelty, however, to our knowledge, no one has used atmospheric chemistry data measured approximately 36 times per day for 20 years in an attempt to map ocean biogeochemistry. Indeed, many have used such data to look at ENSO on a site-by-site basis, but again mapping these across the Pacific and linking to OMZs is new to our knowledge. We are unfortunately confused by the statement made by the reviewer that at the end one cannot identify spatial and temporal heterogeneity as promised because we feel that is exactly what our maps do, and is summarized in Fig. 5. We have included a number of additional lines (please see below) that discuss mechanisms behind our observations as well as some of the limitations of this approach compared with in situ studies.

On the following, I elaborate on the aspects upon which my assessment is based.

**Main comments**

During the introduction the Authors set sail by correctly identifying the heterogeneity of sources and sinks of N2O in the ocean as a major reason for the current uncertainties in the global budget of this climate-relevant trace gas. Likewise, as previous studies did, they identify OMZs as major sources of N2O and suggest the need of correctly attributing the source of their emissions given they disproportionally high role in the global budget given their size, and the fact that they are not easily accessed. Based on this setting, the manuscript (starting with its title) hints to a large-scale temporal and spatial analysis of N2O in the South Pacific. However, beyond air masses passing by an underlying OMZ or not, I would not say the study captures the cross-shelf variability of the coastal emissions, which is the most important feature of the spatial distribution of N2O in the Eastern Tropical South Pacific (ETSP), as much as it is for similar eastern boundary systems (see e.g. Morgan et al., 2015; Paulmier et al., 2008; Farías et al., 2015; references 8 and 15 of the manuscript). Understandably, due to the method used in this study, the large variability along the coast, as well as the seasonal changes (more important for the highly seasonal upwelling off Chile), are smoothed out. However, given the large role of sub-mesoscale variability in setting the quite strong heterogeneity in the biogeochemical environment along the whole eastern boundary (see e.g. Stramma et al., 2013; Thomsen et al., 2015; Köhn et al., 2017), its omission should be at least justified. Moreover, although temporal variability is mentioned in the title and the time series produced by the authors would certainly allow for a detailed analysis, there is little mention to whether or not there is any sign of relevant variability over the last two decades. This is an aspect of the time series which might have been interesting for a wider scientific community, especially in light of recent work showing a global increase in N2O emissions for a comparable time frame as in this study (1998-2016; see Thompson et al., 2019).

We too hypothesized that these analyses should highlight the OMZs, but that hypothesis need not have been correct. A great number of ship-based measurements have produced excellent, high quality data along their cruise tracks, but they do not have global or near-continuous coverage. As the reviewer adeptly points out, cross-shelf variability is of huge importance to localized N_2_O emissions, even within the OMZs. In addition to the excellent references pointed out by the reviewer which we know well, we also attribute much of shelf-based sources to the pioneering work of Wajih Naqvi and his decades of research in the Arabian Sea. Unfortunately, such a detailed attribution is impossible from our data given our 5 degree grid size. The Samoa station is not ideally positioned to be able to resolve such fine-scale spatial resolution in the eastern Pacific with any reasonable certainty, and so we leave the roles of shelves and their variability to the excellent ship-based approaches cited by the Reviewer. For this reason, we did not attempt to describe this variability, and in our aim to be cautious, we omitted any discussion of their role. This now strikes us as an oversight, so we include the sentence, “**Narrow, shallow shelves within the OMZs in particular are important producers of nitrous oxide [Naqvi et al. 2000; Farías et al. 2015; Arévalo-Martínez et al. 2015, Morgan et al. 2019], but local sources with such spatial resolution cannot be identified via our methods.**” [**line 177**]

Similarly, the role of individual sub-mesoscale eddies cannot be identified by using long-term datasets creating an ensemble view of the ocean. Instead, any effects of individual, yet repeated, eddies are averaged among the data. We add the following sentence in this regard: “**We also cannot directly attribute the role of transient sub-mesoscale eddies on enhanced N_2_O production [Altabet et al. 2012, Stramma et al. 2013; Bourbonnais et al. 2015]. While not individually observable from this dataset, such effects are encompassed in the ensemble average over the 20 years of measurements.**” [**line 271**]

In terms of an anthropogenic signal of variability, we were cautious against using these data to prove (or not) the effects of a potentially expanding OMZ on N_2_O production, per Codispoti 2010, Stramma et al. 2008, Schmidtko et al. 2017, among others. Our methods intentionally eliminated the net anthropogenic rise of N_2_O in an effort to look at natural variability/heterogeneity. That said, we agree this is an important area of research to explore, and now include the sentence, “**The effects on these regions of anthropogenic climate change, and whether N_2_O from the OMZs indeed will increase into the future, remain open questions.**” [**line 261**]

Still, at we are able see large-scale features and trends associated with ENSO. We have modified the last sentence of the main text to better highlight this fact: “Nonetheless, the high frequency data from Samoa reveal not only **that** the ETSP **is** a large source of atmospheric N_2_O, but also **that it is** subject to such large changes in short term budgets by ENSO that they are visible **more than half-way** across the Pacific Ocean.” [**line 273**]

On page 5 (Section: *“AGAGE data for marine N2O studies”*) the authors introduce their approach as a tool to *“investigate the role of the OMZs in N2O emission compared with the open ocean”*. Although tracking the influence of N2O emissions from known hotspots (like the ETSP) in the atmospheric mixing ratios of a large oceanic region is intriguing, especially if based on high-quality data as such provided by AGAGE, the scientific question posed by the authors is fundamentally incorrect, since the OMZ extends from the coast to areas well beyond the shelf, which indeed correspond to the open ocean. Thus, a valid comparison would be the close-coastal OMZ vs. the open ocean OMZ and not the coast vs. the OMZ. This misconception becomes problematic when defining the marine region to compare with the atmospheric measurements (see comments below). The authors use an impressive 20-year record of atmospheric mixing ratios of N2O. However, little-to-no analysis and discussion as to whether there are any observable trends over the last two decades is included in the manuscript. Should there be no observable trends, it would be appropriate to mention it since the focus of the paper is not only on spatial but also temporal variability. Moreover, being one of the major points of the manuscript to discuss ENSO-driven modulation of N2O emissions in the South Pacific, it seems odd that little discussion was done on the year 2015, which is the most recent El Niño event and counts as one of the strongest since the 50’s (see e.g. Stramma et al., 2016).

Here we were simplifying “open ocean” to mean the non-OMZ bounded ocean, not to separate the narrow coastal shelf from deep water. We do agree that the impressive quality and length of the AGAGE record has enabled this analysis, and we moreover agree a systematic rise from a changing climate is of primary concern, but that is not the focus of this paper. The reviewer points out a problem, however, in that they anticipated from the title that we would discuss climate change. For this reason, we have opted to amend the title: “On the **natural** spatio-temporal variability of South Pacific nitrous oxide.” Regarding the 2015 El Niño, indeed this was a substantial event, and it is partially included as our data span to October 2015. We realize there was an oversight on our part, omitting this event when listing such events because it was not fully captured among the data, but which we now include on **Line 203**.

Regarding the method associated with the back-trajectory calculations, the authors state that *“It is unclear how far back in time the trajectories remain valid before inaccuracies lead to severe divergences between the calculated path and the actual, and both errors from the climatological data and from the HYSPLIT model itself must be considered in evaluating the certainty of the trajectories”*. However, in the manuscript there is no mention of any attempt to quantify the magnitude of these errors, how they might impact the analysis, and ultimately, the conclusions of the study. Instead, the authors state that *“with the large number of trajectories calculated, emergent patterns indicate any error to be random such that its effect can be minimized”*. This being a study heavily reliant on atmospheric modelling, and the HYSPLIT model being a well- established tool for the purpose intended in this study, I would have expected a more rigorous (quantitative) approach to address this issue. I think it is important to clarify which are the potential sources of inaccuracies in the trajectories and to show if indeed most of them can be safely assumed to be random.

The reviewer is spot on, and it is for this very reason on the error associated with long back trajectories that we gridded our results only in 5-degree bins and do not interpret the data grid cell-by-grid cell. Generally speaking, the error on any individual trajectory is around 20% of its length, but the error on an ensemble is dramatically less, e.g., Freitag et al. *Atmos. Meas. Tech.* (2014). This ensemble clustering is well established for interpreting trace gas measurements, e.g., Cape et al. *Atmos. Envi.* (2000). We have now included these references in this section, and revised the sentences in question to be more explicit: “**Generally, HYSPLIT trajectories incur a distance error on order of 20% of the track length [Freitag et al. 2014], and both errors from the climatological data and from the HYSPLIT model itself must be considered in evaluating the certainty of the trajectories. However, a large number of trajectories calculated within an ensemble, as conducted here, can minimize the influence of any individual track [Cape et al. 2000], and the emergent patterns indicate any error to be random such that its net effect can be minimized.**” [**line 320**].

On pages 6-8 (l.145-182; Section: *“Oxygen minimum zones appear as N2O hotspots”*) the authors aim to relate the results derived from the analysis of atmospheric measurements of N2O mixing ratios and modelled back trajectories to the OMZ in the eastern tropical Pacific. It is intriguing that the authors opt for suggest that OMZs “appear” to be sources of N2O because this is already well-known since the earlier studies from the 70’s (e.g. Elkins et al., 1978). On page 7 (l.154-156) the Authors indicate that their analysis aims to separate biological signals of the tropical Pacific from atmospheric transport originated in the northern hemisphere. However, in this analysis the influence of equatorial upwelling was not considered. Open ocean (equatorial) upwelling is a well-known oceanographic feature of the tropical Pacific and Atlantic oceans, during which significant shoaling and cooling of the mixed layer results in diffusive transport and mixing of chemical tracers including gases like N2O (see e.g. Cline et al., 1987 for the Pacific) to the surface. While this is a strongly seasonal process, the extent of the area with increased sea-air exchange is considerable (in particular in the Pacific) and therefore its effect in the atmospheric budget should be considered. This is relevant not only because of the size of the basin but also because it is precisely in the tropics that one would expect fast transport of trace gases emitted at the surface ocean to upper layers of the atmosphere via convection (see e.g. Xie et al., 2005). Part of the trajectories calculated by the Authors pass by the equatorial region, but in the manuscript there is no mention/discussion on how this may be relevant for their estimates. Although one might argue that the equatorial band indeed lays on top of the OMZ in the tropical Pacific, the source of the waters that rise to the surface during equatorial upwelling are expected to be detached to the portion of the OMZ considered in this study (i.e. waters on the density surface θ = 1026.5 kg m^-3^; see next paragraph of this document).

Equatorial emissions are an important feature, and these surface waters certainly tend to exhibit high dissolved N_2_O concentrations. We absolutely agree the tropics, and equatorial upwelling, are important routes for trace gases to be transported to the upper atmosphere. We should have noted these features explicitly within our results, so we thank the reviewer for pointing out the omission! We have now included the sentence, “**Extension of higher N_2_O anomalies along the equator outside of the 20% O_2_ contour also remain visible west to 140º W (Fig. 3), albeit with reduced magnitude, indicative of a separate likely N_2_O source from equatorial upwelling and subsequent outgassing [Cline et al. 1987 and Weiss et al. 1992].**” [**line 169**]

Our use of the word “appear” is not meant to imply surprise or uncertainty in any fashion. The ODZs do appear (are visible) as hotspots in the sense that oxygen contour co-locates with the highest N_2_O. However, due to the vagueness of the word, in potentially implying an unknown origin, we have retitled the section, “Oxygen minimum zones **emerge** as N_2_O hotspots.” [**line 149**]

The Authors opt for defining density surface θ = 1026.5 kg m-3 (200-500 m depth) as an indication of the minimum oxygen concentration based the paper by Karstensen et al. (2008). However it is not clear at all what is the objective basis for selecting this criteria, since Karstensen et al. (2008) actually report a range of σθ = 26.2-26.8 kg m-3 (or θ = 1026.2-1026.8 kg m^-3^) for a depth range of 150-300 m depth based on the GLODAP v2 data set (same used by the Authors in this manuscript). At this point it should be mentioned that the values presented by Karstensen et al. (2008) represent a mean over the whole South Pacific and therefore its use for only for the eastern side of the basin is not appropriate. As shown on the same work by Karstensen and colleagues, ship-based observations at 85°W show that the oxygen minimum can be found at depths of ca. 300-400 m.

A depth range of 26.2–26.8 kg m^-3^ is reported in Karstensen et al. 2008, of which 26.5 is the average. This range, as the reviewer notes, is for the whole of the Pacific, and thus we are able to apply this condition across the entire South Pacific. Indeed if one looks at the distribution of oxygen saturation along these three horizons in the same GLODAP database referenced by the reviewer, the three are largely indistinguishable except for the North Pacific > 30ºN, a region outside our study. As such, the specific density layer chosen has little effect on the results (Fig. below). All three show the same feature by outlining the zones of low oxygen in the eastern Pacific (and Arabian Sea and Bay of Bengal for that matter).

­

We also point out that within the oxygen deficient waters, 26.5 kg m^-3^ tends to be a very good analogue of the center of the region with lowest oxygen and is a typical value of subtropical mode waters [Gruber 2004]. We are also attaching an oxygen section derived from a recent cruise to the Eastern Tropical North Pacific off of Mexico, in which we sailed along 14ºN. In it, one sees that the lowest oxygen concentrations are indeed in alignment with the 26.5 kg m^-3^ layer (noted with the dashed yellow line), but again, no substantive difference results from the choice of 26.2–26.8. These data are published in Evans et al. *Limnology and Oceanography* (2020). This reference has also been added to the sentence in question to justify this density layer, along with a change to the sentence: “… this value is commonly taken in literature **and in practice** to define the depth layer of the O_2_ minimum in the Eastern Pacific…” [**line 369**].

The lack of clarity as to what defines oxygen threshold, and therefore the extent of the OMZ, is exemplified by the statements on pages 3 (l.69-71) and 7 (l.157-160): While on the first one θ = 1026.5 kg m-3 is claimed to be the layer at which the oxygen (concentrations) are the lowest, on the second this same isopycnal is tied to an oxygen saturation of 20%. To illustrate the difference, I plotted σ_θ_ = 26.5 kg m-3 (or θ = 1026.5 kg m^-3^) both at the oxygen minimum and the 20% oxygen saturation using the same data product used by the Authors in this study (Fig. 1 below). As can be seen, using this isopycnal with respect to the oxygen minimum is hardly applicable for the whole South Pacific but rather part of the ETSP only. Based on the discussion presented by the authors one can only assume that they decided to use 20% oxygen saturation to define the extent of the OMZ. However, this should be clearly stated in order to avoid such ambiguities.

We did not intend for these two sentences to seem incongruous. In the first, we state that the lowest oxygen concentrations in the ocean, within OMZs, align along the 26.5 kg m^-3^ isopycnal. In the second, we denote our OMZ boundary on that isopycnal surface as the 20% horizon saturation. We have reworded the second sentence to read: “**In defining the OMZ boundary by the 20% O_2_ saturation contour on the 1026.5 kg m^-3^ potential density surface, the 15-day back trajectories identify higher than average N_2_O among the air parcels passing over the OMZ** (Fig. 3).” [**line 162**]

Nevertheles, a perhaps more critical issue is that the Authors indicate σθ = 26.5 kg m-3 to collocate with the 200 m depth contour (page 15, l.344-348). As shown by a zonal section of temperature and density anomalies between the Equator and 20°S (Fig. 2 below), the 26.5 kg m-3 isopycnal varies significantly across the basin, reaching depths down to 300-400 m depth at locations which encompass the 20% oxygen saturation boundary applied in this study (c.f. Fig. 1 below). In the absence of evidence that the low oxygen threshold used to define the areal extent of the OMZ contributing to emissions of N2O to the atmosphere lies above the thermocline, and/or providing a mechanistic explanation on how subsurface and surface waters are connected, no compelling case can be made for this region to contribute to the emissions.

Regarding our equating of the 26.5 kg m^-3^ density surface to be “on the order of 200 m below the sea surface”, that was a choice to aid the general audience as few besides oceanographers are used to thinking of marine isopycnal surfaces. Indeed the reviewer is correct, that this layer equates to ~200–300 m in the eastern Pacific but deepens to ~300–500m toward the west (figure below). Regardless, it was only used as an order of magnitude point of reference rather than any substantive alignment. We have adjusted this sentence to say “on the order of 200 **to 500 m** below the sea surface.” [**line 370**].

Based on the results presented, it seems more likely that the vast majority of the positive N2O anomalies observed at the atmospheric station at Samoa could be associated with emissions from close-coastal areas in the ETSP (coastal OMZ), and not the open ocean OMZ. This indeed hints to a disproportionally high contribution of this region (as well as similar eastern boundary upwelling ecosystems) to the global budget of atmospheric N2O. Yet, this has been shown by previous studies. As pointed out above, seasonal emissions during the upwelling season in the equatorial Pacific might be a non-negligible contribution. On a final note to this section, I noticed that reference 14 was used to refer to the relationship between σθ = 26.5 kg m-3 and the oxygen minimum (page 3, l.69-71). However, this is a report on the calibration and quality control procedures carried out for the data product in which no mention of this relationship is made. I kindly recommend a more careful assessment of which literature items are assigned to particular statements on the manuscript.

We make no claim about the contribution of shelves to our observed signal, but again, the reviewer rightly notes that this could be an important next step. The reviewer presents a potential hypothesis that is certainly worth testing, that the emissions from OMZs can be parsed even further to show the concentration along the exceedingly narrow shelves comprises the majority of emissions. We fully admit our methods here cannot tease out this level of fine-scale detail. The GLODAP reference (No. 14 as identified by the reviewer) is the citation of the data product, not just the analysis that went into its quality control. One can use it, like we have, and as the reviewer has, to investigate the alignment of density layers and oxygen across the global oceans. This is GLODAP’s requested method for citing their dataset.

**Fig. 1** Potential density anomaly (σθ) at the lowest oxygen concentration (left panel) and the 20% oxygen saturation (right panel). σθ = 26.5 kg m-3 is indicated by black solid lines. Data from the Global Ocean Data Analysis Project version 2 (GLODAPv2), accessed on May the 20th 2020 ([https://odv.awi.de/data/ocean/glodap-v22019-bottle- data/](https://odv.awi.de/data/ocean/glodap-v22019-bottle-%20data/)).

**Fig. 2** Zonal mean distribution of temperature along a section between the equator and 20°S in the Pacific. σθ = 26.5 kg m-3 is indicated by the black solid line. Data from the Global Ocean Data Analysis Project version 2 (GLODAPv2), accessed on May the 20th 2020 (https://odv.awi.de/data/ocean/glodap-v22019-bottle-data/).

On pages 8-11 (l.184-1242; Section: *“Further modulation by El Niño and La Niña”*) the Authors aim to relate their atmospheric analysis to ENSO cycles over the period of their study to explore how this forcing affects the emissions of N2O from the South Pacific. Given the large impact of ENSO cycles in the physical and biogeochemical environment in coastal and open ocean ecosystems in the Pacific, it is of course a valid approach to aim for a quantification of the extent of this effect. The authors start this section by claiming that the influence of ENSO cycles on the ETSP is *“qualitatively known”*. This statement together with the lack of references, indicates an inexplicable omission of previous work which indeed quantified several aspects of the physical, chemical, and biological oceanography of the region under the influence of El Niño and La Niña events (some examples are Stramma et al., 2016; Yang et al., 2017; Ji et al., 2019; Espinoza-Morriberón et al., 2017; 2019). Unfortunately (and although probably not intended), this comes across as if the authors were trying to oversell their results. The analysis presented by the authors clearly shows ENSO-driven decrease (increase) of N2O emissions from the ETSP during El Niño (La Niña) events. However, beyond this there is no clear discussion as to what this means for basin-scale variability and its relevance in a longer term perspective. Moreover, given that a recent publication (Ji et al., 2019) specifically looked at the production and emissions of N2O in relation to well-known ENSO indexes, reaching the same conclusion presented in this study, also here there is a lack of novelty. Again, although it is an impressive record of atmospheric measurements, a more thorough analysis would be needed to provide new knowledge on the influence of this essential mode of variability on the emissions of N2O.

We appreciate that the reviewer finds our approach valid. We did not intend to slight the excellent work upon which we have built our view of ocean dynamics and the role of ENSO in modulating both OMZs and possible N_2_O production. Certainly we make no intention of claiming how El Niño affects Peruvian upwelling is unknown. However, all of the papers cited by the reviewer either talk about broader features of the effects of ENSO events, or are modeling studies, or include limited ship-based measurements. We are further gratified that the reviewer finds our analysis of ENSO-driven effects clear as this result was far from a certainty when we began the project. We have changed the sentence to read: “It is **~~qualitatively~~** known that ETSP **biogeochemistry** is influenced by the El Niño/La Niña oscillations of the tropical Pacific [Stramma et al. 2016; Espinoza-Morriberón et al. 2017]” [**line 195**].

We also now include the sentence, “**The Pacific-wide decrease in N_2_O observed in the AGAGE back-trajectory analysis is in line with ship-based observations from the eastern [Ji et al. 2019], central [Cline et al. 1987], and western [Butler et al. 1989] Pacific basins.**” [**line 230**]

At the end of the manuscript the Authors conclude: *“Nonetheless, the high frequency data from Samoa reveal not only the ETSP as a large source of atmospheric N2O but also one subject to such large changes in short term budgets by ENSO that they are visible across the Pacific Ocean”*. Again, although an intriguing approach with potential applications to improve the emissions from remote areas for which the data coverage is sparse, in this case the finding that the ETSP is a large source of atmospheric N2O is not a novel one.

The reviewer is correct that other oceanographers have suggested N_2_O sources to be enhanced during La Niña, but this information has not percolated through a broader community. Our hope is that we highlighted this aspect within our paper.

**Additional comments**

Although generally speaking the manuscript is well written, adherence to a coherent format for notating chemical compounds (e.g. nitrous oxide and oxygen) is recommended.

P.3 l.60-61:*“which can rapidly produce N2O because N2O is an obligate metabolic intermediate^8^”*. Being Ref. 8 an excellent manuscript, it is not the most appropriate reference to support that statement.

The reviewer is correct; we have changed the citation to be the seminal work of Zumft et al. 1997. [**line 63**]

P.4 l.91-92:*“N2O exhibits low residence times and rapid turnover rates, priming these areas to display high variability, both over seasonal and interannual scales^8^”*. This manuscript briefly discusses what the results might mean on a wider temporal frame. However it does not directly show any seasonal or interanual variability. I would suggest a more appropriate reference here.

The reference was in regard to residence times and turnover rates. We have now also included the suggested citations of Farías et al. 2015 and Yang et al. 2020. [**line 95**]

P.5-6 l.120-128: I am fully aware than an extensive methodological explanation should not be part of the manuscript. However the formulation confuses the reader since the different stations are discussed without previous mention. Here it would be useful to reformulate the paragraph to briefly explain that four stations were considered, but the station at Samoa ended up being the most suited for the intended analysis.

This is a great idea, thank you. The following sentence was added: “**Multiple stations were considered for analysis, but eliminated as being ill-suited for identifying features in the OMZs.**” [**line 122**].

P.8 l.185-190: References 8 and 12 are great papers. However, their usage in this context is inappropriate since denitrification and N2O production during La Niña events (l.186-188) was not the topic of reference 12 (only mentioned during the final part of the discussion), and reference 8 is not about El Niño events and N2O. Perhaps this is just a formulation issue, but I do suggest the authors to more carefully check for this in order to avoid confusion.

The citation of 12 refers to the organic matter enhancing denitrification rates, which is precisely what that paper was about. We have eliminated the reference with the second point. [**line 200**]

P.7 l.151-152:*“Given the velocity of the trade winds and the distance between Samoa and the ETSP, this time scale is reasonable”*. I would suggest to substantiate such statements better by showing numbers, plots, etc.. Otherwise they may appear as rather vague.

Thank you. We have now included the order of magnitude details: distance is ~10^4^ km with an approximate speed of 15 knots. “Given the velocity of the trade winds and the distance between Samoa and the ETSP, **approx. 8 m s^-1^ and 10^4^ km, respectively,** this time scale is reasonable.” [**line 156**]

P.15 l.342-350: *“Gridding marine oxygen concentrations”*. Considering the weight of this parameter on the study, a more thorough recall on the methods for data gridding and associated uncertainties would be appropriate.

We refer the reviewer to the World Ocean Atlas documentation. This globally-used resource is harnessed across countless publications.

**Reviewer comments, second version:**

Reviewer #1 (Remarks to the Author):

Re-Review of Babbin et al ‘On the spatio-temporal heterogeneity of South Pacific nitrous oxide’

Babbin et al describe in their high-resolution monitoring of atmospheric nitrous oxide (N2O) concentrations in the context of El Niño and La Niña dynamics. Increased concentrations of N2O were connected to La Niña events, and decreased N2O to El Niño. The manuscript has been seen by three reviewers, including myself. All reviewers agreed that the manuscript is of interest to a broad community of scientists, although the reviewers asked for different corrections and changes. Those changes have been made, the open questions of the reviewers, especially mine, have been addressed and therefore I remain with my opinion that this is an important and valuable study, which provides insights to me that have not been shown that clearly elsewhere (so I claim this is novel), the analysis remains convincing, and I recommend this manuscript for publication in Nature Communications.


Reviewer #2 (Remarks to the Author):

Since this is my fourth pass through this paper (!), I will be brief in my remarks. First, I am satisfied with the authors’ response to my own comments. I also carefully reviewed the comments of Reviewer 3 and the authors’ responses. The reviewer made a number of important points, which helped improve the paper and refine the choice of citations. I agree with the reviewer that extensive oceanographic work has been done in the ETSP and that the reduction of the ETSP N2O source during El Niño has been well documented. However, I think that the authors have adequately acknowledged that work, especially in this revised version and thanks to the reviewer’s comments. I disagree that the paper is not novel, since it is the first to use AGAGE atmospheric data and a back trajectory analysis to confirm the importance of a the ETSP OMZ region as a source of N2O and to spatially map the response of that source to ENSO-driven modulations.
One lingering point on which I agree with Reviewer 3 is the conspicuous absence of analysis of the historic 2015-2016 El Niño event in this paper, given the paper’s strong emphasis on ENSO cycles. AGAGE data are publicly available through September 2018, and, with 2 AGAGE coauthors, are probably available even more recently to the study authors. It’s not clear why the study would need to end in October 2015, which misses a substantial part of that El Niño event, which extended through at least March 2016. I appreciate that the HYSPLIT back trajectories may be time consuming to run and would not insist at this late date that the whole analysis be redone (since the authors have already responded in detail to an exceptional level of scrutiny). However, given that it is now 2020, I think the 2015-2016 El Niño event deserves more than just one small mention on line 203.
Overall I think this is an interesting, innovative and well written paper. I would be satisfied to recommend publication with perhaps a new sentence or two commenting on whether the 2015-2016 event appears consistent with the rest of the analysis (i.e., had a suppressed N2O source) or whether it departed substantially from the normal pattern.


Reviewer #3 (Remarks to the Author):

In reviewing an earlier version of this manuscript I pointed out its potential relevance for the field in that it tackles critical issues in our efforts for constraining global budgets of N2O. Despite the high-quality data and analysis used, I expressed my concerns with regards to the way it was presented in terms of its novelty, as well as the lack of clarity with respect to the methods used for oceanographic analysis and how they were used to support author’s conclusions. Having read the revised version submitted by the authors, it is my opinion that most issues raised were satisfactorily addressed and the strengths and limitations of the study are now clearly stated. However, I would still recommend the authors to clarify in the methods section their choice of isopycnal surface for the oxygen analysis. Although this was explained in the response letter, this sort of information should be also provided to any reader of the manuscript. Likewise, and considering that the issue of selecting an oxygen threshold to define the OMZ’s minimum has been clarified now, I still think the manuscript needs to clearly show evidence of a connection between the low-oxygen layer at 200-500 m depth (that constitutes the OMZ’s minimum) and surface waters (where gas exchange with the atmosphere takes place). Spatial co-location between an enhanced atmospheric N2O signal and the underlying, expectedly high-N2O waters at mid-water depth, does not necessarily means that there is causality behind it. In other words, in the absence of a process that creates mixing or advection of N2O-rich waters towards waters in contact with the atmosphere within a reasonably time frame, it is hard to grasp why this is the case. I do not want to contend that this cannot happen but rather that it has to be shown; given the extensive oceanographic data set on hand, this should be doable. I therefore kindly urge the authors to address this issue. As I pointed out during my first review, the approach used in this study has the potential to offer a useful tool for constraining the N2O emissions from remote regions. This is particularly relevant when one considers open ocean regions where slight, yet relevant, changes can be seen only over decades. However, since other researchers might want to try the same approach, its full validity should be demonstrated. Hence, it is important that the authors provide a mechanistic explanation on how the open South Pacific Ocean alters air masses along their way to the west. In my opinion this aspect should be addressed by the authors before their manuscript is published.

**Author rebuttal, second version:**

**Reviewer #1**

Re-Review of Babbin et al ‘On the spatio-temporal heterogeneity of South Pacific nitrous oxide’

Babbin et al describe in their high-resolution monitoring of atmospheric nitrous oxide (N2O) concentrations in the context of El Niño and La Niña dynamics. Increased concentrations of N2O were connected to La Niña events, and decreased N2O to El Niño. The manuscript has been seen by three reviewers, including myself. All reviewers agreed that the manuscript is of interest to a broad community of scientists, although the reviewers asked for different corrections and changes. Those changes have been made, the open questions of the reviewers, especially mine, have been addressed and therefore I remain with my opinion that this is an important and valuable study, which provides insights to me that have not been shown that clearly elsewhere (so I claim this is novel), the analysis remains convincing, and I recommend this manuscript for publication in Nature Communications.

We remain grateful to the reviewer for the effort in thoroughly reviewing our manuscript and providing this feedback.

**Reviewer #2**

Since this is my fourth pass through this paper (!), I will be brief in my remarks. First, I am satisfied with the authors’ response to my own comments. I also carefully reviewed the comments of Reviewer 3 and the authors’ responses. The reviewer made a number of important points, which helped improve the paper and refine the choice of citations. I agree with the reviewer that extensive oceanographic work has been done in the ETSP and that the reduction of the ETSP N2O source during El Niño has been well documented. However, I think that the authors have adequately acknowledged that work, especially in this revised version and thanks to the reviewer’s comments. I disagree that the paper is not novel, since it is the first to use AGAGE atmospheric data and a back trajectory analysis to confirm the importance of a the ETSP OMZ region as a source of N2O and to spatially map the response of that source to ENSO-driven modulations.

That this reviewer has dedicated so much time to our manuscript is extremely appreciated. Thank you again for finding the paper novel and important, and certainly for improving the study and its relevance.

One lingering point on which I agree with Reviewer 3 is the conspicuous absence of analysis of the historic 2015-2016 El Niño event in this paper, given the paper’s strong emphasis on ENSO cycles. AGAGE data are publicly available through September 2018, and, with 2 AGAGE coauthors, are probably available even more recently to the study authors. It’s not clear why the study would need to end in October 2015, which misses a substantial part of that El Niño event, which extended through at least March 2016. I appreciate that the HYSPLIT back trajectories may be time consuming to run and would not insist at this late date that the whole analysis be redone (since the authors have already responded in detail to an exceptional level of scrutiny). However, given that it is now 2020, I think the 2015-2016 El Niño event deserves more than just one small mention on line 203.

This is a good idea; our original dataset used all of the publicly-available data when we started our analyses in summer 2017. We have now extended the dataset through the end of 2016 to fully capture the 2015-2016 El Niño event as requested. The results do not substantively change outside our previous error measurements. This is now identified appropriately throughout the manuscript, notably at line 210: “(1997/98, 2002/03, 2009/10, 2015**/16**)”

Overall I think this is an interesting, innovative and well written paper. I would be satisfied to recommend publication with perhaps a new sentence or two commenting on whether the 2015-2016 event appears consistent with the rest of the analysis (i.e., had a suppressed N2O source) or whether it departed substantially from the normal pattern.

Thank you! We are indebted to your efforts improving this manuscript. The 2015-2016 El Niño event did not produce a substantial change in the data. The 2015-2016 El Niño also exhibited a suppressed OMZ N_2_O source relative to the overall neutral mean. This is now included at Line 236: “The effects are even more pronounced during the strongest of El Niño and La Niña events**, including the recent 2015-2016 El Niño,** with greater shifts from the mean state during the most intense periods (Fig. 5).”

**Reviewer #3**

In reviewing an earlier version of this manuscript I pointed out its potential relevance for the field in that it tackles critical issues in our efforts for constraining global budgets of N2O. Despite the high-quality data and analysis used, I expressed my concerns with regards to the way it was presented in terms of its novelty, as well as the lack of clarity with respect to the methods used for oceanographic analysis and how they were used to support author’s conclusions. Having read the revised version submitted by the authors, it is my opinion that most issues raised were satisfactorily addressed and the strengths and limitations of the study are now clearly stated.

Thank you again for your thorough reading and recommendations for improving the manuscript.

However, I would still recommend the authors to clarify in the methods section their choice of isopycnal surface for the oxygen analysis. Although this was explained in the response letter, this sort of information should be also provided to any reader of the manuscript. Likewise, and considering that the issue of selecting an oxygen threshold to define the OMZ’s minimum has been clarified now, I still think the manuscript needs to clearly show evidence of a connection between the low-oxygen layer at 200-500 m depth (that constitutes the OMZ’s minimum) and surface waters (where gas exchange with the atmosphere takes place). Spatial co-location between an enhanced atmospheric N2O signal and the underlying, expectedly high-N2O waters at mid-water depth, does not necessarily means that there is causality behind it. In other words, in the absence of a process that creates mixing or advection of N2O-rich waters towards waters in contact with the atmosphere within a reasonably time frame, it is hard to grasp why this is the case. I do not want to contend that this cannot happen but rather that it has to be shown; given the extensive oceanographic data set on hand, this should be doable. I therefore kindly urge the authors to address this issue. As I pointed out during my first review, the approach used in this study has the potential to offer a useful tool for constraining the N2O emissions from remote regions. This is particularly relevant when one considers open ocean regions where slight, yet relevant, changes can be seen only over decades. However, since other researchers might want to try the same approach, its full validity should be demonstrated. Hence, it is important that the authors provide a mechanistic explanation on how the open South Pacific Ocean alters air masses along their way to the west. In my opinion this aspect should be addressed by the authors before their manuscript is published.

We agree these are important non-obvious points. We have added these sentences to the text: **Whereas water this deep does not tend to equilibrate rapidly with the atmosphere, this density surface reflects broader features in the water column, such as the existence of a shallower N_2_O maximum driven by microbial processes^18^. This shallower N_2_O maximum can be more easily transmitted to the surface waters from the interplay of upwelling and lateral and vertical mixing^32^. Mesoscale and sub-mesoscale eddies in particular, below the spatial resolution that can be resolved by these data, are likely pathways that promote mixing of deeper N_2_O into the surface waters^33,34^.** [Lines 166-172].

**Reviewer comments, third version:**

**Author rebuttal, third version:**
